# A Hybrid Flux Balance Analysis and Machine Learning Pipeline Elucidates Metabolic Adaptation in Cyanobacteria

**DOI:** 10.1016/j.isci.2020.101818

**Published:** 2020-11-18

**Authors:** Supreeta Vijayakumar, Pattanathu K.S.M. Rahman, Claudio Angione

**Affiliations:** 1 Department of Computer Science and Information Systems, Teesside University, Middlesbrough, North Yorkshire TS1 3BX, UK; 2 Centre for Enzyme Innovation, Institute of Biological and Biomedical Sciences, School of Biological Sciences, University of Portsmouth, Portsmouth, Hampshire PO1 2UP, UK; 3 Tara Biologics, Woking, Surrey GU21 6BP, UK; 4 Centre for Digital Innovation, Teesside University, Middlesbrough TS1 3BX, UK; 5 Healthcare Innovation Centre, Teesside University, Middlesbrough TS1 3BX, UK

**Keywords:** Metabolic Engineering, In Silico Biology, Artificial Intelligence, Bioengineering

## Abstract

Machine learning has recently emerged as a promising tool for inferring multi-omic relationships in biological systems. At the same time, genome-scale metabolic models (GSMMs) can be integrated with such multi-omic data to refine phenotypic predictions. In this work, we use a multi-omic machine learning pipeline to analyze a GSMM of *Synechococcus* sp. PCC 7002, a cyanobacterium with large potential to produce renewable biofuels. We use regularized flux balance analysis to observe flux response between conditions across photosynthesis and energy metabolism. We then incorporate principal-component analysis, *k*-means clustering, and LASSO regularization to reduce dimensionality and extract key cross-omic features. Our results suggest that combining metabolic modeling with machine learning elucidates mechanisms used by cyanobacteria to cope with fluctuations in light intensity and salinity that cannot be detected using transcriptomics alone. Furthermore, GSMMs introduce critical mechanistic details that improve the performance of omic-based machine learning methods.

## Highlights

• A pipeline for metabolic modeling in *Synechococcus* sp. PCC 7002 is presented
• Metabolic fluxes display clear differences in pathway activity across conditions
• Omic-informed GSMMs provide critical mechanistic details within machine learning
• Combining GSMM and machine learning improves methods based on transcriptomics alone

## Graphical Abstract

**Figure:**
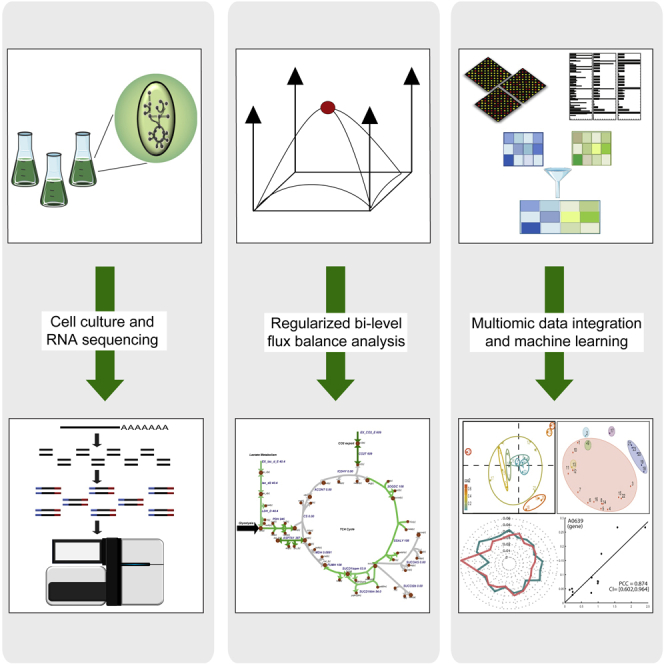

## Introduction

In the field of systems biology, several approaches have been proposed to capture the enormous complexity of biological systems by utilizing mathematical modeling and computational methods, with the goal of amalgamating the information required to build and refine predictive models. The challenges presented by such an undertaking are numerous and persistent owing to the size, format, scale, and variation of the disparate data types. Among these, metabolism is currently the only biological layer that can be modeled genome-wide (O'Brien et al., 2015; Haas et al., 2017). Constraint-based reconstruction and analysis methods are commonly used to express metabolic flux through biochemical reactions based on knowledge of reaction stoichiometry. Flux balance analysis (FBA) is particularly suitable for modeling metabolic networks at the genome scale, as the definition of kinetic parameters and metabolite concentrations is not a key requisite.

In recent years, genome-scale metabolic models (GSMMs) have been integrated with multiple data types, including omics, codon usage, enzyme costs, and limited resource availability (Abedpour and Kollmann, 2015; Opdam et al., 2017; Kashaf et al., 2017; Wortel et al., 2018; Tian and Reed, 2018; Angione, 2019). This serves to exploit the large volume of experimental data being generated from high-throughput omics technologies. In doing so, additional constraints can be applied during FBA to shrink the solution space (Reed, 2012), thus providing a more accurate representation of metabolic capability as a greater number of factors can be considered to explain cellular behavior. This can prove useful in refining phenotypic predictions across various environmental conditions (Vijayakumar et al., 2017; Sánchez et al., 2017; van der Ark et al., 2017; Angione, 2018) and can predict steps to engineer an organism in a way that optimizes the production of certain metabolites, which is highly applicable in many fields of industrial biotechnology including the production of biofuels, biosurfactants, and pharmaceuticals (Angione et al., 2015; Dougherty et al., 2017; Huang et al., 2017; Fatma et al., 2018; Occhipinti et al., 2018).

### Modeling and Metabolic Engineering in Cyanobacteria

*Cyanobacteria* is a phylum of oxygenic, phototrophic microalgae that need to adapt to constant fluctuations in temperature, salinity, light intensity (or irradiance), and nutrient availability, among other factors (Montgomery, 2017; Blanco-Ameijeiras et al., 2018; Gunde-Cimerman et al., 2018). Metabolic engineering is helping to develop cyanobacteria into photoautotrophic biofactories that can act as production hosts (chassis) for alcohols, carbohydrates, organic acids, fatty acid derivatives, isoprenoids, and many other chemicals (Noreña-Caro and Benton, 2018). However, as such approaches are generally designed with heterotrophic organisms in mind, the metabolic features unique to photoautotrophs must be considered, e.g., pathways relating to photosynthesis and CO_2_ fixation (Carroll et al., 2018).

*Synechococcus* sp. PCC 7002 is a fast-growing cyanobacterium that flourishes in both freshwater and marine environments, owing to its ability to tolerate high light intensity and a wide range of salinities. Harnessing the properties of cyanobacteria has become an important goal in recent years owing to their potential to serve as biocatalysts for the production of renewable biofuels (Hendry et al., 2016). Metabolic modeling of two cyanobacteria, *Arthrospira* and *Synechocystis*, has successfully characterized the use of photosynthetic electron transport components in different light conditions (Toyoshima et al., 2020).

In an industrial setting, *Synechococcus* sp. PCC 7002 has been recommended as the ideal chassis for the mass cultivation of microalgae for biotechnological applications owing to its ease of genetic manipulation as well as its tolerance for high salinity, light intensity, and temperature (Pade and Hagemann, 2014; Clark et al., 2018). These are highly desirable traits in microalgae as they enable cultures to maintain a rapid growth rate in open raceway ponds as well as in photobioreactors, which operate at high temperatures (Ruffing et al., 2016). Within the *Synechococcus* genus, a comparative analysis of slow- and fast-growing strains in terms of their active reactions under phototrophic conditions has been proposed to better inform their development into production hosts (i.e., strain optimization), primarily through maximizing their growth rates (Hendry et al., 2019). In a recent study, Song et al. (2015) completed an integrative analysis of metabolic and gene co-expression networks in *Synechococcus* sp. PCC 7002 by integrating expression data from either continuous cultures or existing studies into a GSMM and deriving fluxes using E-Fmin flux minimization (Song et al., 2014) and MOMA (Segre et al., 2002). Further studies have examined temporal variations in response to varying light intensity and associated conditional dependencies (Rügen et al., 2015; Reimers et al., 2016). These need to be accounted for as constraints in GSMMs designed to simulate the phototrophic growth in cyanobacteria over diurnal cycles and tackle issues associated with resource allocation (Vijayakumar and Angione, 2017).

Genome-scale isotopic non-stationary metabolic flux analysis (INST-MFA) has been utilized to estimate internal metabolic fluxes more accurately in *Synechococcus elongatus* UTEX 2973, toward the aim of establishing factors affecting phototrophic metabolism under optimal growth conditions (Hendry et al., 2019). Similarly, MOMA and INST-13C MFA were used to establish carbon partitioning at intracellular branching points in the central metabolism of a glycogen-deficient *Synechococcus* sp. PCC 7002 mutant (Hendry et al., 2017). Such models benefit significantly from constraints designed using experimentally measured uptake or growth rates for the identification of alternative reactions responsible for the synthesis of metabolites and differences in pathway recruitment and utilization (e.g., for carbon conversion to biomass).

The current state of strain-specific metabolic modeling in cyanobacteria, and the potential of fluxomic data and metabolic engineering, have been recently discussed elsewhere (Angermayr et al., 2015; Oliver et al., 2016; Hendry et al., 2020; Luan et al., 2020; Babele and Young, 2020; Mukherjee et al., 2020; Hitchcock et al., 2020). Within the *Synechococcus* genus, a comparative analysis of slow- and fast-growing strains in terms of their active reactions under phototrophic conditions has been proposed to better inform their development into production hosts (i.e., strain optimization), primarily by maximizing their growth rates (Hendry et al., 2019). A number of novel, non-model strains of *Synechococcus* that have been developed include *Synechococcus* UTEX 2973 (Yu et al., 2015), PCC 11801 (Jaiswal et al., 2018), PCC 11802 (Damini et al., 2020), PCC 11901 (Włodarczyk et al., 2020), and BDU 130192 (Ahmad et al., 2020).

### Multi-omic Data Integration in Microalgae

In recent years, synthetic biology has facilitated the modeling of biological processes for genetic engineering. Using synthetic biology tools, algal strains have been designed according to highly specific environmental conditions and yield requirements. Synthetic biologists have been successful in assembling genetic material and manipulating the lipid content of microalgae, as well as maximizing biomass accumulation and biofuel yield (Jagadevan et al., 2018). These results are promising for the biofuels industry from the microalgal perspective (Randhawa et al., 2017). In the context of microalgae, the alteration of lipid biosynthesis pathways through the induction of a stress response to a change in environment (such as temperature, nutrient limitation, salinity) is a common practice to enhance the production of target compounds, including those that are used to produce workable biofuels (Rawat et al., 2013).

Omics approaches have made a significant contribution to the understanding of the molecular processes of microalgae. Furthermore, the discoveries that omics studies have made, e.g., the identification of genes involved in specific processes, may be vital to the engineering of enhanced microalgae. Through the understanding of transcription levels and gene activation data gathered from transcriptomics, the effectiveness of genetic alterations can be measured as previously achieved for other organisms, allowing for optimization of the target product. For example, if the new gene insert is operating at its optimum, the transcriptomic data should show an increase in the mRNA of the target gene when compared with the wild type (Randhawa et al., 2017). Based on genomic and transcriptomic data, Wang et al. (2019) recently identified a series of neutral sites on the chromosome of *Synechococcus* sp. PCC 7002 for the introduction of novel heterologous genes or pathways without disruption.

Omics techniques can also provide valuable insights into alterations of lipid synthesis pathways that occur as a result of stress conditions in microalgae. Metabolomics studies assess the low-molecular-weight metabolic end products and are highly indicative of response to stresses. Previously, global transcriptomic, proteomic, and metabolomic analyses have aided in identifying adaptations for cyanobacterial salt tolerance in *Synechocystis* sp. PCC 6803 (Pandhal et al., 2009; Wang et al., 2016). An omic-combination approach would allow for optimization of algal engineering, as the data gathered from transcriptomics should show an increase in transcription in the gene of interest that coincides with a reduction in metabolism caused by stress (such as nutrient limitation) highlighted by metabolomics, if the expression of the gene of interest is linked to a metabolic process. The application of omic studies can not only ascertain the effectiveness of any genetic modification but also be used to optimize the scale-up process. With the use of spatial and temporal omics studies of systems such as raceways used for algal growth, a deeper understanding of how algae will perform in various areas of the raceway can be gained, allowing for process optimization (Randhawa et al., 2017).

### Aims and Objectives

**Figure 1:**
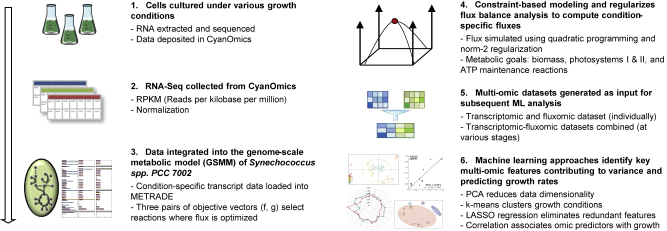
A Multi-omic Machine Learning Pipeline for Prediction and Classification of *Synechococcus* Metabolic Features (1) RNA sequencing data obtained from *Synechococcus* sp. PCC 7002 cells grown under 23 growth conditions (Ludwig and Bryant, 2011, 2012a,b). (2) Data downloaded from CyanOmics (Yang et al., 2015). (3) Starting from a model recently published by Hendry et al. (2016), the condition-specific GSMMs of *Synechococcus* sp. PCC 7002 are generated by integrating omics data, and three pairs of objectives are optimized for each condition-specific model. (4) Bilevel regularized FBA is conducted using quadratic programming to compute regularized flux distributions. (5) Transcriptomic, fluxomic, and multi-omic (combination) datasets are preprocessed for the machine learning analysis. (6) PCA, *k*-means clustering, LASSO regression, and correlation analysis are applied to identify latent cross-omic patterns in the metabolic adaptation mechanisms. These techniques are applied and compared across three sets of omic features: gene transcripts, condition-specific flux rates, and a combination of both omics.

In this work, we present a pipeline combining metabolic modeling with statistical and machine learning tools (Figure 1) for analyzing a GSMM of the cyanobacterium *Synechococcus* sp. PCC 7002. We characterize *Synechococcus* adaptation mechanisms using an updated GSMM of *iSyp702* containing 728 genes (Hendry et al., 2016), implementing multi-objective FBA with quadratic regularization. We then apply machine learning techniques to identify functionally important genes and reactions. These include PCA, *k*-means clustering, and LASSO regression, which serve not only to identify biologically significant gene transcripts and fluxes but also to relate these features more effectively to growth-promoting or growth-limiting conditions provided by the initial expression data.

Our goal is to show whether, in a predictive setting, features derived from the metabolic model can add meaningful information to the features derived from the transcriptomic data (Zampieri et al., 2019). Therefore, for each method, we will consider the predictions yielded using three sets of features: (1) gene expression only, (2) fluxes only, and (3) gene expression and fluxes combined.

Through LASSO regression, we find that using flux rates to predict growth rates is more effective than using gene transcript values alone. This suggests that GSMMs provide critical details in terms of stoichiometry, and the involvement of genes in reactions determines the rate of cellular phototrophic growth as well as other modes of energy utilization (e.g., heterotrophy, mixotrophy) in various environmental conditions.

## Results

**Figure 2:**
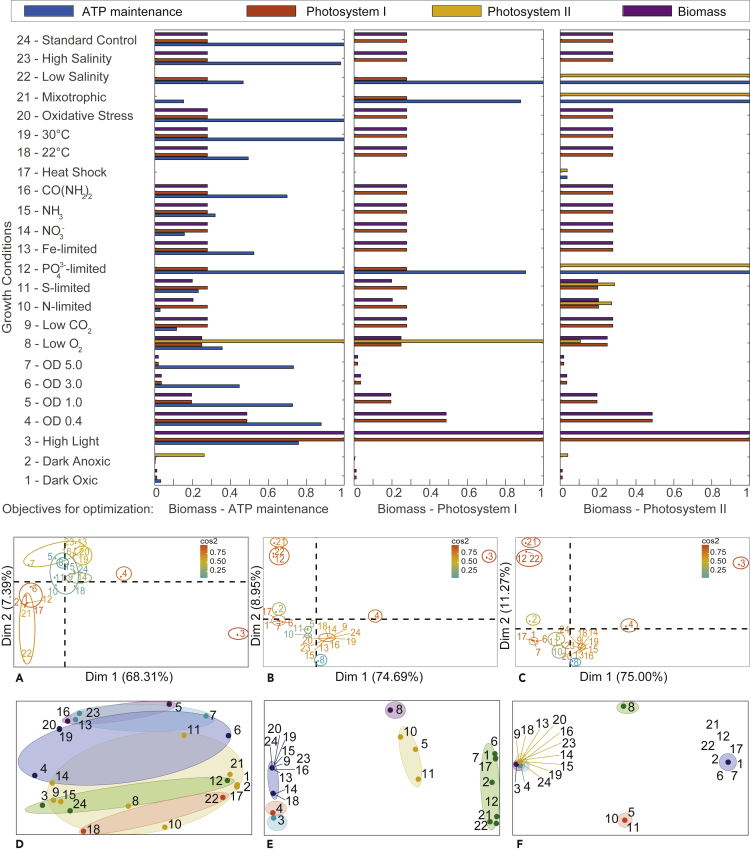
Flux Distributions with Fluxomic PCA and *k*-means (Top): Flux distributions in the 24 growth conditions considered in this study. Flux distributions for four key reactions: ATP maintenance, photosystem I, photosystem II, and biomass when running FBA using three different pairs of objectives (indicated at the bottom of each plot). Conditions 1–24 correspond to those detailed in Table S1. To better visualize the differences in flux between conditions, flux values were normalized by dividing by the maximal flux (i.e., the flux value for the control condition) for that reaction across all conditions. The full list of flux rates is reported in the Supplemental Information. Regularized FBA correctly predicts reduced growth in sub-optimal conditions and the highest biomass flux is given by the high light condition. (Bottom): Fluxomic principal-component analysis (PCA) and *k*-means. PCA (A–C) and *k*-means clustering (panels D–F) were conducted using the entire flux distribution (742 reactions). (A) and (D) are associated with Biomass-ATP maintenance fluxes, (B) and (E) with Biomass-Photosystem I fluxes, and (C) and (F) with Biomass-Photosystem II fluxes. For PCA plots, growth conditions are colored according to their cos2 value, which indicates the contribution of the first two components to the squared distance of each condition to the origin (Abdi and Williams, 2010). The higher the cos2 value, the greater the proportion of contribution to the total distance, meaning that the importance of the principal components is greater for that condition. For *k*-means, data are clustered by condition (where the colors of ellipses represent different clusters) and the number of clusters (*k*=6) was selected following silhouette analysis. Due to co-location of conditions in the two-dimensional plot, not all overlapping points are visible, but the cluster associated with each condition is labeled. The full list of growth conditions and their respective *k*-means clusters are reported in the Supplemental Information.

**Figure 3:**
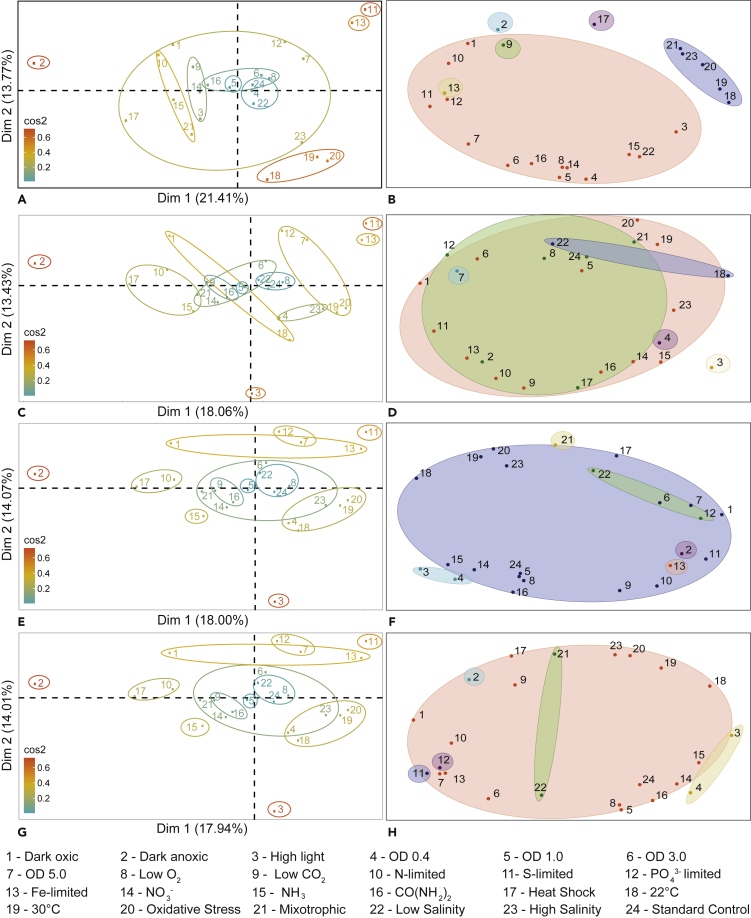
Transcriptomic and Multi-omic Principal-Component Analysis (PCA) and *k*-means PCA and *k*-means clustering conducted for 3,187 gene transcripts (A and B) and both transcripts and fluxes, with Biomass-ATP maintenance (C and D), Biomass-Photosystem I (E and F), and Biomass-Photosystem II (G and H) as objective pairs. In the PCA plots (A, C, E, and G), growth conditions are colored according to their cos2 value, indicating the contribution of the first two components to the squared distance of each condition to the origin (Abdi and Williams, 2010). For *k-*means, data are clustered by condition (where the color of the ellipses represents different clusters) and the number of clusters was determined following silhouette analysis (*k*=6). When compared to using the transcriptomic dataset alone, the combined proportion of variance for PCA in the first two dimensions was slightly higher when gene transcript data was used in isolation than when it was combined with fluxes. For *k*-means clustering, the change in objective pair used for FBA did not result in a significant difference in the clusters formed. However, there is a clear demarcation between clusters of conditions that limit growth (e.g., low light, sulfate limitation) and those that promote growth (e.g., high light, nitrate supplementation).

**Figure 4:**
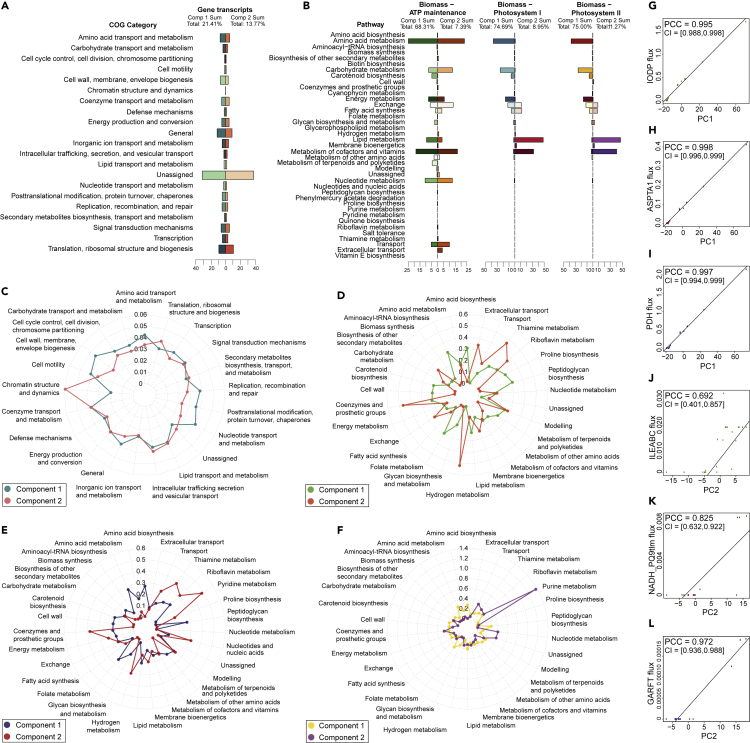
Pathway-Based PCA to Identify Pathway and Reaction Contribution to Variance across Conditions (A and B) Component sums by pathway. Principal component contributions summed across reactions within each COG category/pathway, to decompose the metabolic function of the main contributors to variance. The total percentage of contribution to variance of the first two principal components is also given for each dataset: (A) gene transcripts and (B) fluxes calculated with objective functions Biomass-ATP maintenance, Biomass-Photosystem I, and Biomass-Photosystem II. (C–F) Component contribution by pathway. Average principal component contributions within each pathway, calculated across all gene transcripts (C) and fluxes for each objective function pair: (D) Biomass-ATP maintenance, (E) Biomass-Photosystem I, and (F) Biomass-Photosystem II. (G–L) Interpreting PCA coordinates with fluxes. The Pearson correlation coefficient (PCC) was calculated between the principal component coordinates and the flux values across the conditions in three pairs of objectives. The coordinates for the first principal component (*x* axis) and flux (*y* axis) across the 24 conditions were plotted for the following reactions: (G) inorganic diphosphatase (IODP) for Biomass-ATP maintenance, (H) aspartate transaminase (ASPTA1) for Biomass-Photosystem I, and (I) pyruvate dehydrogenase (PDH) for Biomass-Photosystem II. For the second principal component, the reactions were: (J) L-isoleucine transport via ABC system (ILEABC) for Biomass-ATP maintenance, (K) NADH dehydrogenase type II in the thylakoid membrane (NADH_PQ9tlm) for Biomass-Photosystem I, and (L) phosphoribosylglycinamide formyltransferase (GARFT) for Biomass-Photosystem II. The PCC with their respective 95% confidence intervals (CI) are displayed within each plot.

**Figure 5:**
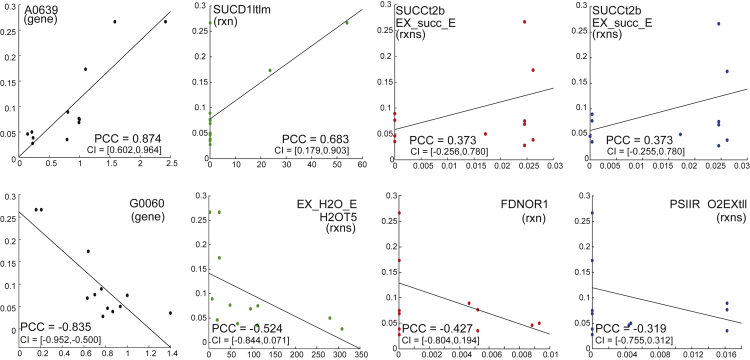
Top PCCs with Their Respective 95% Confidence Intervals (CI) between Gene Transcript/Reaction Flux Data (*x* axis) and Growth Rates (*y* axis) Left to right: Black, top correlated genes; green, top correlated reactions when maximizing Biomass-ATP maintenance flux; red, top correlated reactions when maximizing Biomass-Photosystem I flux; blue, top correlated reactions when maximizing Biomass-Photosystem II flux. Tables S9–S13 list the top 10 genes/reactions in the dataset that are positively or negatively correlated with growth and their respective PCC values. Additional figures of top 10 genes/reactions and their respective PCC values are provided in the Supplemental Information (Figures S1–S4).

**Figure 6:**
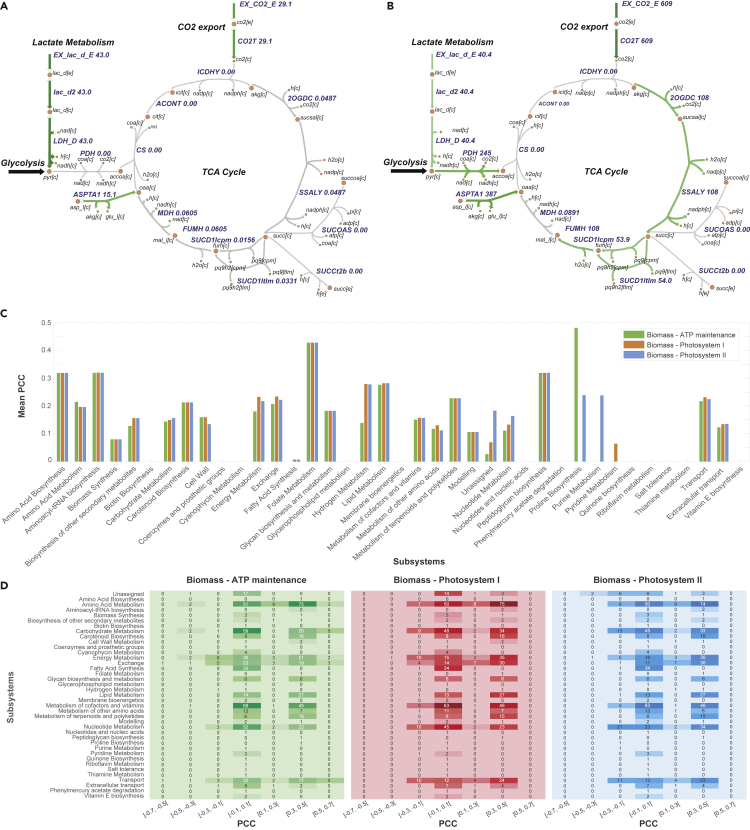
Flux Map Comparison and PCC Values by Subsystem Comparison between flux values in the TCA cycle for (A) nitrogen limitation and (B) urea supplementation. The SUCD1Itlm and SUCD1Icpm reactions encoding succinate dehydrogenase were identified as having a strong positive correlation with the growth rate for the Biomass-ATP maintenance objective pair. (C) Mean absolute Pearson correlation coefficient (PCC) values calculated between 12 experimental growth rates and their corresponding condition-specific GSMM reaction fluxes within each metabolic subsystem/pathway in the *Synechococcus* sp. PCC 7002 GSMM. The highest mean absolute correlations were identified for folate metabolism, proline, and amino acid biosynthesis. (D) Reactions within each model subsystem sorted into classes of PCC values obtained between growth rates and flux rates in each objective pair (Biomass-ATP maintenance, Biomass-Photosystem I, Biomass-Photosystem II). The Biomass-ATP maintenance pair yielded the highest positive PCC values [0.5, 0.7[ for reactions within the carbohydrate, amino acid, energy, transport, and exchange metabolic pathways.

As highlighted above, our goal is to reconnect metabolism to growth and other cellular objectives using a data-driven multi-view approach that yields biologically reasonable predictions. The results of PCA and *k*-means clustering for flux data are included in Figure 2, whereas results of these analyses for gene transcript data in isolation and gene transcript data combined with fluxes are detailed in Figure 3. Additionally, the results of the pathway-wide analysis of principal components are provided in Figure 4. The highest positive/negative Pearson correlation coefficient (PCC) values for transcript- and flux-only datasets are given in Figure 5, whereas mean absolute PCCs for metabolic subsystems or pathways are shown in Figure 6C with the number of reactions in each subsystem specified in Figure 6D. A list of all nonzero LASSO coefficients and the top 10 positive/negative correlation coefficients are given in the Supplemental Information, with the full calculation of these coefficients provided in Data S2. An interpretation of the results for each technique used is provided below.

### Regularized Flux Balance Analysis

Compared with transcriptomics, metabolic flux data modeled at the genome-scale provide a more comprehensive, condition-specific view of the phenotype. Therefore, we mapped each RNA sequencing profile measured in 24 growth conditions to a *Synechococcus* GSMM, and we employed a regularized FBA to obtain condition-specific flux distributions (see Transparent Methods in Supplemental Information). To calculate the flux rates more accurately for each condition, several lower and upper bounds were adjusted before performing FBA, according to specific growth media and other requirements described for each growth condition (Ludwig and Bryant, 2011, 2012a, 2012b). The full details of these growth conditions (including composition of growth media, optical density at the time of cell harvestation, mode of energy utilization, availability of oxygen/carbon dioxide, light intensity, salinity, and temperature) are listed in Table S1. The full specification of constraints for each growth condition is given in Data S3.

From the transcriptomic studies listed in Table S1, there were a number of genes that were not transcribed in the control condition but were transcribed specifically under perturbed conditions. Many of these genes have yet to be assigned a particular functional category or encode hypothetical proteins, but many more have been linked to specific pathways and compounds and some have been associated with the adaptation of *Synechococcus* sp. PCC 7002 to atypical environmental or growth conditions.

As shown in Figure 2, apart from the standard control, the highest fluxes through the ATP maintenance reaction (when ATP maintenance is set as the secondary objective) were among conditions that limit growth, such as phosphate limitation, 30^∘^C, and oxidative stress. In dark anoxic, low-salinity, heat shock, phosphate limitation, and mixotrophic conditions, there was no flux for the biomass reaction. However, for all the objective pairs, the flux through the biomass during high light intensity and OD 0.4 (optical density) was higher than the control condition (0.19 mmol gDW^−1^ h^−1^ and 0.093 mmol gDW^−1^ h^−1^ respectively, compared with 0.053 mmol gDW^−1^ h^−1^). The biomass is likely to be higher at OD 0.4 than OD 0.7 due to adjustment of the photon constraint, which allowed for more transmission of light at lower OD. Apart from the dark anoxic and low O_2_ conditions, all fluxes through photosystem II were negligible, but the fluxes through photosystem I were still maintained (0.058 mmol gDW^−1^ h^−1^ in the high light intensity condition as opposed to 0.016 mmol gDW^−1^ h^−1^ in the control).

When photosystem I was set as the secondary objective, a low amount of flux through ATP maintenance reaction was retained in phosphate-limited, heat shock, and low salinity conditions (approximately 0.0002 mmol gDW^−1^ h^−1^). When photosystem II was set as the secondary objective, the highest fluxes through the photosystem II reaction were given by the phosphate limitation, mixotrophic, and low salinity conditions (0.016 mmol gDW^−1^ h^−1^).

Lack of light is likely to be the greatest contributing factor to decrease in growth as low oxygen concentration does not seem to stunt growth, as the proportional decrease in biomass was lower relative to the standard control conditions. On the other hand, there appears to be little to no flux for the biomass or the photosystem I reaction in the dark conditions. This is supported by Vu et al. (2013), who reported that lower yields under dark conditions may be due to the limited generation of energy (ATP) and reductant (NADPH) from glycogen in the absence of photoautotrophic growth. When optical density was varied through the batch growth conditions (OD 0.4, 1.0, 3.0, and 5.0), the transmission of light through the cultures decreased as the dry cell weight (DCW) increased. Equal reduction in transcript levels for the photosynthetic apparatus was previously observed in all macronutrient-limited conditions studied (Ludwig and Bryant, 2012a). We infer that heat shock, mixotrophic growth, and phosphate limitation have the largest effect on reducing growth rate, as there was a complete impairment of biomass production predicted across all of our objectives for these conditions. This is in line with reported findings (Ludwig and Bryant, 2012a), where perturbations caused by phosphate limitation had a greater impact on the global transcription pattern than observed for high irradiance or dark treatments.

*Synechococcus* sp. PCC 7002 is known to possess one of the greatest tolerances for high light intensity among cyanobacteria (with an upper limit of approximately 2,000 μmol photons m^−2^ s^−1^) (Xiong et al., 2015). This was evident from our predictions for all three pairs of objectives, where flux through the biomass pathway during high light intensity was slightly higher than the control condition (0.192 mmol gDW^−1^ h^−1^ compared with 0.053 mmol gDW^−1^ h^−1^). Although the fluxes through photosystem II were disrupted, the fluxes through photosystem I were still maintained (0.058 mmol gDW^−1^ h^−1^ in the high light intensity condition as opposed to 0.016 mmol gDW^−1^ h^−1^ in the control). Heat shock resulted in no fluxes through any of the four reactions within all three objective pairs. It was previously reported that transcript levels for genes encoding photosystem I decreased slightly in cells grown at high salinity and remained constant at low salinity (Ludwig and Bryant, 2012b). On the other hand, it was found that transcript levels for genes encoding photosystem II did not change in response to fluctuations in salinity (Ludwig and Bryant, 2012b).

### Multi-omic Principal-Component Analysis

It can be argued that analyzing single-omic data alone has limited relevance in the context of metabolic processes, as it does not capture the full complexity of the phenotype in relation to environmental variability. The hybrid approach proposed in this work connects transcriptomic and fluxomic data using a data-driven multi-view approach that supports machine learning algorithms to yield more accurate predictions (Culley et al., 2020). Considering the vast dimensionality of multi-omic models, the identification of biologically meaningful information can prove to be challenging. As a non-parametric statistical technique, principal-component analysis (PCA) was incorporated into our workflow for identifying patterns and genes/reactions responsible for the most variance in the datasets (Brunk et al., 2016).

The PCA indicates the proportion of variance exhibited by fluxes in the first two dimensions for each objective pair. For all three pairs of objectives in Figures 2A–2C, over 68.31% of the variance can be explained by the first dimension when considering flux data alone. As shown in plot (a), the high light intensity condition contributed the highest score for the first dimension, accounting for the vast majority of the variance. On the other hand, plots (b) and (c) showed that high light intensity, phosphate limitation, mixotrophic, and low salinity were the highest scoring conditions in the first dimension. For the second dimension, the highest score was given by high salinity, iron limitation, urea, 30^∘^C and oxidative stress in plot (a), and high light intensity, phosphate limitation, mixotrophic, and low salinity conditions in plots (b) and (c).

When considering only the gene transcript data (Figure 3A), the combined proportion of variance that could be accounted for by the first two dimensions was vastly reduced (only 35.18% compared with 75.70%–86.27% for flux data). The conditions with the largest scores for the first dimension were sulfate and iron limitation, followed by oxidative stress, 30^∘^C, and high salinity. Once again, sulfate and iron limitation were the highest in the second dimension, along with phosphate limitation, nitrogen limitation, dark anoxic/oxic, and the last phase of the batch growth (OD 5.0).

When using a combined dataset of both gene transcripts and fluxes (Figures 3C, 3E, and 3G), the total proportion of variance that could be explained in two dimensions for all three objective pairs was lower than using transcript data alone (31.43%–32.07%). The highest scores were given by iron and sulfate limitation in the first dimension and by dark oxic, dark anoxic, OD 5.0, iron limitation, and sulfate limitation in the second dimension.

A full list of gene transcript and calculated fluxes are included in Data S1. For a list of the top 10 contributions of genes and reactions to the principal components, we refer the reader to the Supplemental Information (Tables S3–S5).

#### Pathway-Level Analysis of Principal Components

To further examine the most metabolically significant pathways or cellular processes, we also performed a pathway-level PCA while categorizing genes and reaction by their main function. Owing to the varying number of reactions within each pathway, both the pathway sum and average contribution to the variance from the first two principal components were calculated. In Figures 4A and 4B, the sum of all contributions to variance within each pathway or COG (Cluster of Orthologous Groups) category is summarized. For the gene transcripts (Figure 4A), the COGs with the highest sum of variance within the first two principal components were poorly characterized (with general or unknown function). It can be observed that for each pair of flux objectives in (Figure 4B), the pathways that contribute the most to the first and second components were similar: cofactor and vitamin metabolism, nucleotide metabolism, energy metabolism, lipid metabolism, amino acid metabolism, carbohydrate metabolism, and transport metabolism. These pathways can be directly linked to cellular growth because many of their products are biomass precursors or compounds that can be catabolized to produce energy, i.e., carbohydrates, proteins, and fats.

The radar plots in Figures 4C–4F depict the average contributions to the variance within each pathway for the first and second principal components. The average contribution was higher for reactions (fluxes) than genes because the number of genes in each COG category was greater than the number of reactions in each subsystem of the GSMM. The pathways with the highest average contributions for gene transcripts were nucleotide/amino acid metabolism in the first component and chromatin structure and dynamics in the second component. For all three objective pairs, amino acid, aminoacyl-tRNA, and peptidoglycan biosyntheses were relevant in the first component (with an average contribution between 0.26 and 0.3). For the second component, the pathways with the largest contribution varied for each objective. Coenzyme and thiamine metabolism both had an average contribution greater than 0.45 for the second component in relation to ATP and photosystem I fluxes, with the addition of hydrogen metabolism for ATP and pyridine metabolism for photosystem I. On the other hand, purine and nucleotide metabolism had the highest contributions in the second component for photosystem II. However, many of these pathways contained only one or two reactions, which caused these results to be skewed; for example, the purine metabolism pathway had an average contribution of 1.36, but only contains one nucleotide phosphodiesterase reaction (PDE2).

Finally, to characterize the PCA in the context of single reactions, we analyzed the principal component coordinates for all growth conditions against different reaction fluxes selected from the top 10 contributors to the variance in each of the three objective pairs (see Figures 4G–4L). These plots confirmed that a large part of the variance could be explained in the first principal component, as the first component coordinates showed a near-perfect correlation with flux (> 0.99). The second principal component displayed a less consistent but still strongly positive correlation between coordinates and flux values. The set of reactions with the highest contributions to variance in the first and second components were completely different, but three main functional categories could be identified among these reactions. IODP and GARFT can be linked to nucleotide metabolism, ASPTA1 and ILEABC to amino acid metabolism, and PDH and NADH_PQ9tlm to energy metabolism.

### Clustering

*k*-means is a clustering algorithm that computes clusters while iteratively minimizing the sum of squared Euclidean distances between each observation and its respective cluster mean (McLachlan et al., 2008). To assess whether the generated multi-omic datasets could identify clusters of growth conditions according to the respective omic responses, we applied *k*-means to the set of 24 growth conditions, considering gene expression, flux rates, and the combined expression/flux dataset.

For the flux data (plots d–f in Figure 2), the partitioning of *k*-means clusters varied depending on the pair of FBA objectives for which fluxes were calculated. Following silhouette analysis, the number of clusters set for plots (d–f) in Figures 2, 3B, 3D, 3F, and 3H was *k*=6. The full list of members of each cluster is reported in the Supplemental Information.

When combining both transcript and flux data (Figure 3D, 3F, and 3H), the clusters formed were less distinct. This suggests that fluxes could help to contribute more biological insights into metabolic reactions (through the metabolic network) that are not available in the transcriptomic data. Nevertheless, through the *k*-means analysis with transcripts-only and the combined multi-omic dataset of transcripts and fluxes, we conclude that clustering techniques benefit from analyzing the flux and transcript datasets in isolation rather than combining them, as this avoids an increase in data dimensionality that cannot be easily reduced.

In most of the *k*-means plots in Figure 3, changing the objective pair used for FBA did not result in a significant difference in the clusters formed. However, there was sometimes a demarcation between clusters of conditions that limited growth (e.g., low light, nutrient limitation) and those that promoted growth (e.g., high light availability, nutrient supplementation). In some instances, certain conditions (e.g., heat shock, high light intensity, iron limitation) were isolated within a single cluster. Furthermore, reducing the number of dimensions in the data following PCA could serve to reduce noise and make the definition of clusters even clearer.

### LASSO Regression

Regression-based algorithms can ascertain a mapping function, given a number of continuous output variables *y* and a number of real-valued or discrete input variables *x*. For each input variable, a coefficient is estimated with linear regression, determining the importance of the input variable toward predicting the output variable. In our study, the least absolute shrinkage and selection operator (LASSO) was used to select a subset of the input variables by minimizing the number of nonzero coefficients. It employs L1 regularization, which penalizes the sum of absolute values of all the coefficients; this sets the coefficients of unnecessary or recursive features equal to zero, resulting in a sparser matrix (Tibshirani, 1996). The regularization enables the identification of important predictors, elimination of redundant predictions, and generation of shrinkage estimates with lower predictive errors.

The LASSO regression identified genes and reactions in the model that are strongly related to *in vivo* growth rates through the retention of non-zero predictor coefficients (see Table S2). The full calculation of LASSO coefficients is provided in Data S2.

A complete list of non-zero predictors retained by LASSO for each dataset is given in the Supplemental Information. The functional classifications were provided by CyanOmics in the case of genes (CY Category and CY Sub Category) or the *subsystems* field within the model GSMM for reactions. In Tables S6 and S8 (where both transcript and flux datasets or only the gene transcripts are considered), the same non-zero predictors (genes) were retained, irrespective of the objective pair used for FBA. The genes yielding positive coefficients were associated with photosynthesis and respiration or post-translational modification of proteins. When applying the LASSO algorithm to the flux-only dataset (see Table S7), the non-zero coefficients retained were primarily related to the metabolism of nucleotides, cofactors, and vitamins and pathways relating to energy generation, such as carbohydrate and amino acid metabolic pathways. Such co-factors are often composed of metal ions, for which there are numerous transport and exchange reactions, e.g., cobalt and manganese.

### Correlation Analysis

The Pearson correlation coefficients were calculated to ascertain the strength of the association between transcripts and/or flux rates and growth across different conditions (see Table S2). The absolute Pearson correlation coefficients were sorted in descending order, and the top 10 positive/negative correlation coefficients for each dataset are listed in the Supplemental Information. Figure 5 shows the highest positive and negative Pearson correlation coefficients for transcript- and flux-only datasets. The gene A0639 encodes a phycocyanin-associated phycobilisome rod-core linker polypeptide, which is an important component of the photosynthetic apparatus. This confirms that photosynthesis and energy metabolism are directly correlated with cellular growth.

For the Biomass-ATP maintenance flux objectives, the selection of reactions encoding succinate dehydrogenase in the cytoplasmic and thylakoid membranes (SUCD1Icpm and SUCD1Itlm) demonstrated the importance of the tricarboxylic acid (TCA) cycle in the generation of energy for biomass accumulation. To illustrate this, a comparison of flux values between the nitrogen-limited and urea-supplemented growth conditions is provided in Figures 6A and 6B. It can be seen in Table S2 that the limitation or supplementation of a nitrogen source had a direct effect on the growth rate. Cyanobacteria have long been known to possess a unique TCA cycle where an alternative reaction homologous to 2OGDH is used to convert alpha-ketoglutarate into succinyl semialdehyde (2OGDC), which is subsequently converted into succinate (via succinate-semialdehyde dehydrogenase, i.e., SSALY) (Zhang and Bryant, 2011; Steinhauser et al., 2012). 2OGDC and SSALY were found to carry negligible flux under phototrophic conditions in (Hendry et al., 2016), which was supported by the flux values derived for the standard control for our simulations (3.599 mmol gDW^−1^ h^−1^ for both reactions), as well as the growth-limiting conditions such as nitrogen limitation (Figure 6A). This suggests that succinate dehydrogenation plays an important role in growth; it is known to still take place in dark, anoxic conditions (McNeely et al., 2010) where there is an increased flux toward succinate during the dark period, driving ATP production through respiratory electron transport (Sarkar et al., 2019).

Figure 6C shows the mean absolute PCC values among reactions within each subsystem, whereas Figure 6D shows the number of reactions within a given range of PCC values for each subsystem/pathway listed in the model, to account for the differing number of reactions in each pathway in the model. The pathway with the largest mean absolute correlation across all the flux objectives was the folate metabolism. In *Synechocystis* sp. PCC 6803, folate is synthesized from chorismate and is known to be important for cellular processes such as DNA replication, repair, and methylation, in addition to being a vital precursor for the biosynthesis of certain amino acids, co-factors, nucleotides, and tRNAs (Mills et al., 2020). The highest mean PCC in the biomass-ATP maintenance flux pair corresponded with the proline biosynthesis pathway/reaction. Interestingly, it has been found that proline accumulation is highly induced in stress conditions in cyanobacteria, especially high salinity (increased NaCl concentration) because it plays a role in osmoprotection, antioxidative defense, and signaling (Hayat et al., 2012; Pingkhanont et al., 2019). Across all flux objectives, the majority of reactions in Figure 6D have a correlation value between [-0.1, 0.1[ or [0.3, 0.5[, implying most reactions had little to no significant correlation or a moderately positive correlation. The strongest positive correlation values [0.5, 0.7[ were found between the Biomass-ATP maintenance flux pair and the growth rates. It can be seen that these reactions were classified under various pathways, i.e., carbohydrate, amino acid, energy, and transport- and exchange-related metabolism.

## Discussion

In this work, we showed how using a hybrid multi-view approach with multi-omic data and machine learning to yield metabolically significant fluxes enabled the identification of trends in data that were not apparent using solely transcriptomic data. We used condition-specific FBA to obtain flux distributions with L2-regularized bilevel optimization.

The flux distributions obtained for four key reactions showed clear differences in pathway activity across the various conditions and also between the three pairs of objectives used. When comparing the results across the types of datasets used, it is clear that complex metabolic and phenotypic outcomes as a result of adaptation to a changing environment are difficult to predict from gene expression alone. Condition-specific metabolic models within a machine learning framework allowed for the detection of coordinated responses shared between different data types, as well as the variation in responses across different growth conditions. Although a large number of studies express the maximization of biomass as the only objective when performing FBA, it is imperative to recognize that in reality most organisms have multiple objectives to satisfy. It has been well established that the activity of biosynthetic and energy-generating pathways increases with the growth rate (Bernstein et al., 2014), which led us to implement multi-level regularized optimization in our pipeline, considering more than one objective function.

Specifically, when calculating the flux distribution across conditions, biomass was chosen as the primary objective, whereas the secondary objective was set to ATP maintenance, photosystem I, or photosystem II, to reflect the main cellular goals of cyanobacteria. Biomass was chosen as a primary objective to represent the maximization of growth rate and cellular yields (Feist and Palsson, 2010; Yuan et al., 2016; Lakshmanan et al., 2019), which is a critical consideration for the production of biofuels by cyanobacteria as this informs the substrate uptake rates and maintenance requirements that indicate fundamental cellular growth requirements. The chosen secondary objectives are key pathways involved in energy metabolism during photosynthesis. Simulating the cost of ATP maintenance can help to examine the energy required for sustaining metabolic activity even in the absence of growth. The incorporation of the photoexcitation reactions occurring within photosystems I and II served to characterize how flux under various conditions reflects the light harvesting and energy transfer via photon absorption through these complexes. Thus, solving the quadratic optimization problem for multiple pairs of objectives helped to resolve trade-offs by considering the conditions and constraints affecting each of these objectives (Sajitz-Hermstein and Nikoloski, 2016; Occhipinti et al., 2020).

Our results suggest that it is worth using model-generated flux data that incorporates transcriptomics to conduct machine learning analyses. The flux data were initially informed by transcriptomic data as the condition-specific gene expression profiles were generated by combining them with a baseline GSMM for *Synechococcus* sp. PCC 7002; in this way, gene transcripts already constituted an important component of the FBA. Furthermore, reducing the number of dimensions in the data following PCA can serve to reduce noise and make the definition of clusters even clearer. In addition to this, a reduced set of predictors were identified as being related to growth as a result of the LASSO regularization. Specifically, the identification of reactions by LASSO as key features, which are of potential use for the prediction of growth rates, supports the inclusion of metabolic fluxes as features for future applications of regression techniques with smaller, more concise sets of flux data.

The reactions identified as being strongly correlated with the growth rate in the flux datasets (SUCD1Itlm, SUCD1Icpm, ME2) suggest that fluxes can help to gain more biological insights into machine learning analyses. As a different, unrelated set of genes displayed a strong correlation with the growth rate, it is evident that analyzing both transcriptomic and fluxomic data provides a more complete picture of cyanobacterial metabolism than single-omic analyses. In particular, the role of metal transport pathways in cyanobacteria was significant because they are highly relevant in the context of photosynthesis. The detection of latent, biologically significant patterns and adaptive mechanisms to fluctuations in light intensity and salinity elucidates the maintenance of metabolic efficiency at the cellular level, as well as the attainment of multiple cellular objectives.

Algal engineering supplemented with data from multi-omic studies can contribute to informing the scale-up of these processes. Such multi-omic data are sensitive enough to detect the effect of stress on metabolism. Metabolic engineers could apply this pipeline to test more strategies *in silico* when developing the optimal production host, or to analyze multi-omic outputs (both independently and in combination with other omic data). In this regard, the use of transcriptomic data to characterize fluxomic predictions elucidates many of the unique mechanisms employed by *Synechococcus* sp. PCC 7002 when adapting to changes in light intensity, salinity, and other conditions. In the case of cyanobacteria, we also emphasize the importance of assessing model inputs in accordance with specific growth conditions before conducting FBA. These contribute to the organism's underlying objective of maintaining metabolic efficiency for phototrophic growth and light-dependent photosynthesis. As a result of predicting and classifying metabolic profiles in various growth conditions, our approach sheds light on the cross-omic mechanisms of its adaptation process, which enables survival across a wide range of environments and stress conditions.

### Limitations of the Study

The availability of exact measurements for the various growth conditions could yield more precise flux predictions. For example, in our case, the exact photon absorbance of the *Synechococcus* sp. PCC 7002 cultures was not available. Hence, the photon uptake constraints were approximated using DCW and photon consumption based on the availability of light. Likewise, the setting of nutrient uptake rates was approximated based on data provided by *in vivo* experiments rather than measured directly.

Furthermore, in this study we adopted linear transformations and linear methods, where possible. This was with the goal of maximizing the biological interpretability of the predictions, using quadratic terms for regularization only. However, different dimensionality reduction or clustering methods could be implemented, e.g., to elucidate any further non-linear relationship among the omic elements.

Finally, there is further potential for other types of omic data to be integrated into the model (e.g., from proteomic or metabolomic datasets). It is expected that integrating further omic datasets, i.e., further data views in our multi-view machine learning setting, could produce even more detailed insights into metabolic adaptations, or better support existing findings derived from transcriptomic and fluxomic data.

### Resource Availability

#### Lead Contact

Dr. Claudio Angione, email: c.angione@tees.ac.uk.

#### Materials Availability

The study did not generate new unique reagents or other materials.

#### Data and Code Availability

The complete source code of our pipeline is freely available on GitHub at https://github.com/Angione-Lab/Synechococcus7002-metabolic-modelling.

The RNA-seq data integrated into the model were from transcriptomic datasets uploaded by Ludwig and Bryant (2011, 2012a, 2012b). These were acquired directly from the Cyanomics dataset (Yang et al., 2015), but have since been made available on the NCBI Sequence Read Archive. The accession numbers for the RNA-seq data reported in this paper are SRA:SRP007372, SRA:SRP013965 and SRA:SRP066851.

## Methods

All methods used in this study can be found in the accompanying Transparent Methods section in the Supplemental Information file.

## Supplemental Information

Document S1. Transparent Methods, Figures S1–S4, and Tables S1–S13

Data S1. Full Transcript and Flux Data, Related to Figures 2 and 3 Microsoft Excel workbook containing RPKM transcript reads for each growth condition, fold changes calculated for transcripts, complete flux rates for three objective pairs, and fold changes calculated for flux rates.

Data S2. Growth Rates, LASSO, and Pearson Correlation, Related to Figure 5 Microsoft Excel workbook containing: (1) a list of 24 growth conditions with growth rates and doubling times calculated for 11 conditions relative to cultures grown under standard conditions; (2) fitted least-squares regression beta coefficients and mean squared errors for LASSO conducted with gene transcripts and/or flux rates and growth rates; (3) Pearson correlation coefficients calculated between gene transcripts and/or flux rates and growth rates listed with p values, lower and upper bounds according to the 95% confidence interval, and coefficients of determination (R^2^); (4) mean absolute Pearson correlation coefficients within each group of reactions in each subsystem of the GSMM.

Data S3. Photon Unit Conversions and Flux Constraints, Related to Figure 2 Microsoft Excel workbook containing (1) calculation of photon uptake rate for FBA in different growth rates and (2) full list of modified flux constraints (model.lb and model.ub) used to perform FBA.

## Footnotes

Supplemental Information can be found online at https://doi.org/10.1016/j.isci.2020.101818.

## References

[bib1] Abdi H., Williams L.J. (2010). Principal component analysis. Wiley Interdiscip. Rev. Comput. Stat..

[bib2] Abedpour N., Kollmann M. (2015). Resource constrained flux balance analysis predicts selective pressure on the global structure of metabolic networks. BMC Syst. Biol..

[bib3] Ahmad A., Pathania R., Srivastava S. (2020). Biochemical characteristics and a genome-scale metabolic model of an indian euryhaline cyanobacterium with high polyglucan content. Metabolites.

[bib4] Angermayr S.A., Rovira A.G., Hellingwerf K.J. (2015). Metabolic engineering of cyanobacteria for the synthesis of commodity products. Trends Biotechnol..

[bib5] Angione C. (2018). Integrating splice-isoform expression into genome-scale models characterizes breast cancer metabolism. Bioinformatics.

[bib6] Angione C. (2019). Human systems biology and metabolic modelling: a review—from disease metabolism to precision medicine. Biomed. Res. Int..

[bib7] Angione C., Costanza J., Carapezza G., Lió P., Nicosia G. (2015). Multi-target analysis and design of mitochondrial metabolism. PLoS One.

[bib8] van der Ark K.C., van Heck R.G., Dos Santos V.A.M., Belzer C., de Vos W.M. (2017). More than just a gut feeling: constraint-based genome-scale metabolic models for predicting functions of human intestinal microbes. Microbiome.

[bib9] Babele P.K., Young J.D. (2020). Applications of stable isotope-based metabolomics and fluxomics toward synthetic biology of cyanobacteria. Wiley Interdiscip. Rev. Syst. Biol. Med..

[bib10] Bernstein H.C., Konopka A., Melnicki M.R., Hill E.A., Kucek L.A., Zhang S., Shen G., Bryant D.A., Beliaev A.S. (2014). Effect of mono-and dichromatic light quality on growth rates and photosynthetic performance of *Synechococcus* sp. pcc 7002. Front. Microbiol..

[bib11] Blanco-Ameijeiras S., Moisset S.A., Trimborn S., Campbell D.A., Heiden J.P., Hassler C.S. (2018). Elemental stoichiometry and photophysiology regulation of *Synechococcus* sp. pcc 7002 under increasing severity of chronic iron limitation. Plant Cell Physiol..

[bib12] Brunk E., George K.W., Alonso-Gutierrez J., Thompson M., Baidoo E., Wang G., Petzold C.J., McCloskey D., Monk J., Yang L. (2016). Characterizing strain variation in engineered e. coli using a multi-omics-based workflow. Cell Syst..

[bib13] Carroll A.L., Case A.E., Zhang A., Atsumi S. (2018). Metabolic engineering tools in model cyanobacteria. Metab. Eng..

[bib14] Clark R.L., McGinley L.L., Purdy H.M., Korosh T.C., Reed J.L., Root T.W., Pfleger B.F. (2018). Light-optimized growth of cyanobacterial cultures: growth phases and productivity of biomass and secreted molecules in light-limited batch growth. Metab. Eng..

[bib15] Culley C., Vijayakumar S., Zampieri G., Angione C. (2020). A mechanism-aware and multiomic machine-learning pipeline characterizes yeast cell growth. Proc. Natl. Acad. Sci. U S A.

[bib16] Damini J., Annesha S., Shinjinee S., Swati M., Pakrasi H.B., Wangikar P.P. (2020). A novel cyanobacterium *Synechococcus elongatus* pcc 11802 has distinct genomic and metabolomic characteristics compared to its neighbor pcc 11801. Sci. Rep..

[bib17] Dougherty B.V., Moutinho T.J., Papin J. (2017).

[bib18] Fatma Z., Hartman H., Poolman M.G., Fell D.A., Srivastava S., Shakeel T., Yazdani S.S. (2018). Model-assisted metabolic engineering of *Escherichia coli* for long chain alkane and alcohol production. Metab. Eng..

[bib19] Feist A.M., Palsson B.O. (2010). The biomass objective function. Curr. Opin. Microbiol..

[bib20] Gunde-Cimerman N., Plemenitaš A., Oren A. (2018). Strategies of adaptation of microorganisms of the three domains of life to high salt concentrations. FEMS Microbiol. Rev..

[bib21] Haas R., Zelezniak A., Iacovacci J., Kamrad S., Townsend S., Ralser M. (2017). Designing and interpreting ‘multi-omic’experiments that may change our understanding of biology. Curr. Opin. Syst. Biol..

[bib22] Hayat S., Hayat Q., Alyemeni M.N., Wani A.S., Pichtel J., Ahmad A. (2012). Role of proline under changing environments: a review. Plant Signal. Behav..

[bib23] Hendry J.I., Bandyopadhyay A., Srinivasan S., Pakrasi H.B., Maranas C.D. (2020). Metabolic model guided strain design of cyanobacteria. Curr. Opin. Biotechnol..

[bib24] Hendry J.I., Gopalakrishnan S., Ungerer J., Pakrasi H.B., Tang Y.J., Maranas C.D. (2019). Genome-scale fluxome of *Synechococcus elongatus* utex 2973 using transient 13c-labeling data. Plant Physiol..

[bib25] Hendry J.I., Prasannan C., Ma F., Möllers K.B., Jaiswal D., Digmurti M., Allen D.K., Frigaard N.U., Dasgupta S., Wangikar P.P. (2017). Rerouting of carbon flux in a glycogen mutant of cyanobacteria assessed via isotopically non-stationary 13c metabolic flux analysis. Biotechnol. Bioeng..

[bib26] Hendry J.I., Prasannan C.B., Joshi A., Dasgupta S., Wangikar P.P. (2016). Metabolic model of *Synechococcus* sp. pcc 7002: prediction of flux distribution and network modification for enhanced biofuel production. Bioresour. Technol..

[bib27] Hitchcock A., Hunter C.N., Canniffe D.P. (2020). Progress and challenges in engineering cyanobacteria as chassis for light-driven biotechnology. Microb. Biotechnol..

[bib28] Huang Z., Lee D.Y., Yoon S. (2017). Quantitative intracellular flux modeling and applications in biotherapeutic development and production using cho cell cultures. Biotechnol. Bioeng..

[bib29] Jagadevan S., Banerjee A., Banerjee C., Guria C., Tiwari R., Baweja M., Shukla P. (2018). Recent developments in synthetic biology and metabolic engineering in microalgae towards biofuel production. Biotechnol. Biofuels.

[bib30] Jaiswal D., Sengupta A., Sohoni S., Sengupta S., Phadnavis A.G., Pakrasi H.B., Wangikar P.P. (2018). Genome features and biochemical characteristics of a robust, fast growing and naturally transformable cyanobacterium *Synechococcus elongatus* pcc 11801 isolated from India. Sci. Rep..

[bib31] Kashaf S.S., Angione C., Lió P. (2017). Making life difficult for clostridium difficile: augmenting the pathogen’s metabolic model with transcriptomic and codon usage data for better therapeutic target characterization. BMC Syst. Biol..

[bib32] Lakshmanan M., Long S., Ang K.S., Lewis N.E., Lee D.Y. (2019). On the impact of biomass composition in constraint-based flux analysis. bioRxiv.

[bib33] Luan G., Zhang S., Lu X. (2020). Engineering cyanobacteria chassis cells toward more efficient photosynthesis. Curr. Opin. Biotechnol..

[bib34] Ludwig M., Bryant D.A. (2011). Transcription profiling of the model cyanobacterium *Synechococcus* sp. strain pcc 7002 by next-gen (solid™) sequencing of cdna. Front. Microbiol..

[bib35] Ludwig M., Bryant D.A. (2012). Acclimation of the global transcriptome of the cyanobacterium *Synechococcus* sp. strain pcc 7002 to nutrient limitations and different nitrogen sources. Front. Microbiol..

[bib36] Ludwig M., Bryant D.A. (2012). *Synechococcus* sp. strain pcc 7002 transcriptome: acclimation to temperature, salinity, oxidative stress, and mixotrophic growth conditions. Front. Microbiol..

[bib37] McLachlan G.J., Bean R.W., Ng S.K., Jonathan M.K. (2008). Clustering. Bioinformatics.

[bib38] McNeely K., Xu Y., Bennette N., Bryant D.A., Dismukes G.C. (2010). Redirecting reductant flux into hydrogen production via metabolic engineering of fermentative carbon metabolism in a cyanobacterium. Appl. Environ. Microbiol..

[bib39] Mills L.A., McCormick A.J., Lea-Smith D.J. (2020). Current knowledge and recent advances in understanding metabolism of the model cyanobacterium *Synechocystis* sp. pcc 6803. Biosci. Rep..

[bib40] Montgomery B.L. (2017). Seeing new light: recent insights into the occurrence and regulation of chromatic acclimation in cyanobacteria. Curr. Opin. Plant Biol..

[bib41] Mukherjee B., Madhu S., Wangikar P.P. (2020). The role of systems biology in developing non-model cyanobacteria as hosts for chemical production. Curr. Opin. Biotechnol..

[bib42] Noreña-Caro D., Benton M.G. (2018). Cyanobacteria as photoautotrophic biofactories of high-value chemicals. J. CO2 Util..

[bib43] O’Brien E.J., Monk J.M., Palsson B.O. (2015). Using genome-scale models to predict biological capabilities. Cell.

[bib44] Occhipinti A., Eyassu F., Rahman T.J., Rahman P.K., Angione C. (2018). In silico engineering of *Pseudomonas* metabolism reveals new biomarkers for increased biosurfactant production. PeerJ.

[bib45] Occhipinti A., Hamadi Y., Kugler H., Wintersteiger C., Yordanov B., Angione C. (2020). Discovering essential multiple gene effects through large scale optimization: an application to human cancer metabolism. IEEE/ACM Trans. Comput. Biol. Bioinform..

[bib46] Oliver N.J., Rabinovitch-Deere C.A., Carroll A.L., Nozzi N.E., Case A.E., Atsumi S. (2016). Cyanobacterial metabolic engineering for biofuel and chemical production. Curr. Opin. Chem. Biol..

[bib47] Opdam S., Richelle A., Kellman B., Li S., Zielinski D.C., Lewis N.E. (2017). A systematic evaluation of methods for tailoring genome-scale metabolic models. Cell Syst..

[bib48] Pade N., Hagemann M. (2014). Salt acclimation of cyanobacteria and their application in biotechnology. Life.

[bib49] Pandhal J., Noirel J., Wright P.C., Biggs C.A. (2009). A systems biology approach to investigate the response of synechocystis sp. pcc6803 to a high salt environment. Saline Syst..

[bib50] Pingkhanont P., Tarasuntisuk S., Hibino T., Kageyama H., Waditee-Sirisattha R. (2019). Expression of a stress-responsive gene cluster for mycosporine-2-glycine confers oxidative stress tolerance in *Synechococcus elongatus* pcc 7942. FEMS Microbiol. Lett..

[bib51] Randhawa K.S., Relph L.E., Armstrong M.C., Rahman P.K. (2017). Biofuel production: tapping into microalgae despite challenges. Biofuels.

[bib52] Rawat I., Kumar R.R., Mutanda T., Bux F. (2013). Biodiesel from microalgae: a critical evaluation from laboratory to large scale production. Appl. Energ..

[bib53] Reed J.L. (2012). Shrinking the metabolic solution space using experimental datasets. PLoS Comput. Biol..

[bib54] Reimers A.M., Knoop H., Bockmayr A., Steuer R. (2016). Evaluating the stoichiometric and energetic constraints of cyanobacterial diurnal growth. arXiv.

[bib55] Ruffing A.M., Jensen T.J., Strickland L.M. (2016). Genetic tools for advancement of *Synechococcus* sp. pcc 7002 as a cyanobacterial chassis. Microb. Cell Fact..

[bib56] Rügen M., Bockmayr A., Steuer R. (2015). Elucidating temporal resource allocation and diurnal dynamics in phototrophic metabolism using conditional fba. Sci. Rep..

[bib57] Sajitz-Hermstein M., Nikoloski Z. (2016). Multi-objective shadow prices point at principles of metabolic regulation. Biosystems.

[bib58] Sánchez B.J., Zhang C., Nilsson A., Lahtvee P.J., Kerkhoven E.J., Nielsen J. (2017). Improving the phenotype predictions of a yeast genome-scale metabolic model by incorporating enzymatic constraints. Mol. Syst. Biol..

[bib59] Sarkar D., Mueller T.J., Liu D., Pakrasi H.B., Maranas C.D. (2019). A diurnal flux balance model of *Synechocystis* sp. pcc 6803 metabolism. PLOS Comput. Biol..

[bib60] Segre D., Vitkup D., Church G.M. (2002). Analysis of optimality in natural and perturbed metabolic networks. Proc. Natl. Acad. Sci. U S A.

[bib61] Song H.S., McClure R.S., Bernstein H.C., Overall C.C., Hill E.A., Beliaev A.S. (2015). Integrated in silico analyses of regulatory and metabolic networks of *Synechococcus* sp. pcc 7002 reveal relationships between gene centrality and essentiality. Life.

[bib62] Song H.S., Reifman J., Wallqvist A. (2014). Prediction of metabolic flux distribution from gene expression data based on the flux minimization principle. PLoS One.

[bib63] Steinhauser D., Fernie A.R., Araújo W.L. (2012). Unusual cyanobacterial tca cycles: not broken just different. Trends Plant Sci..

[bib64] Tian M., Reed J.L. (2018). Integrating proteomic or transcriptomic data into metabolic models using linear bound flux balance analysis. Bioinformatics.

[bib65] Tibshirani R. (1996). Regression shrinkage and selection via the lasso. J. R. Stat. Soc. Ser. B Methodol..

[bib66] Toyoshima M., Toya Y., Shimizu H. (2020). Flux balance analysis of cyanobacteria reveals selective use of photosynthetic electron transport components under different spectral light conditions. Photosynth. Res..

[bib67] Vijayakumar S., Angione C. (2017). Multi-omic data integration elucidates *Synechococcus* adaptation mechanisms to fluctuations in light intensity and salinity. International Conference on Bioinformatics and Biomedical Engineering.

[bib68] Vijayakumar S., Conway M., Lió P., Angione C. (2017). Seeing the wood for the trees: a forest of methods for optimization and omic-network integration in metabolic modelling. Brief. Bioinformatics.

[bib69] Vu T.T., Hill E.A., Kucek L.A., Konopka A.E., Beliaev A.S., Reed J.L. (2013). Computational evaluation of *Synechococcus* sp. pcc 7002 metabolism for chemical production. Biotechnol. J..

[bib70] Wang M., Luan G., Lu X. (2019). Systematic identification of a neutral site on chromosome of *Synechococcus* sp. pcc 7002, a promising photosynthetic chassis strain. J. Biotechnol..

[bib71] Wang Y., Chen L., Zhang W. (2016). Proteomic and metabolomic analyses reveal metabolic responses to 3-hydroxypropionic acid synthesized internally in cyanobacterium synechocystis sp. pcc 6803. Biotechnol. Biofuels.

[bib72] Włodarczyk A., Selão T.T., Norling B., Nixon P.J. (2020). Newly discovered *Synechococcus* sp. PCC 11901 is a robust cyanobacterial strain for high biomass production. Communications Biology.

[bib73] Wortel M.T., Noor E., Ferris M., Bruggeman F.J., Liebermeister W. (2018). Metabolic enzyme cost explains variable trade-offs between microbial growth rate and yield. PLoS Comput. Biol..

[bib74] Xiong Q., Feng J., Li S.t., Zhang G.y., Qiao Z.x., Chen Z., Wu Y., Lin Y., Li T., Ge F. (2015). Integrated transcriptomic and proteomic analysis of the global response of *Synechococcus* to high light stress. Mol. Cell Proteomics.

[bib75] Yang Y., Feng J., Li T., Ge F., Zhao J. (2015). Cyanomics: an integrated database of omics for the model cyanobacterium Synechococcus sp. pcc 7002. Database.

[bib76] Yu J., Liberton M., Cliften P.F., Head R.D., Jacobs J.M., Smith R.D., Koppenaal D.W., Brand J.J., Pakrasi H.B. (2015). *Synechococcus elongatus* utex 2973, a fast growing cyanobacterial chassis for biosynthesis using light and co 2. Sci. Rep..

[bib77] Yuan H., Cheung C., Hilbers P.A., van Riel N.A. (2016). Flux balance analysis of plant metabolism: the effect of biomass composition and model structure on model predictions. Front. Plant Sci..

[bib78] Zampieri G., Vijayakumar S., Yaneske E., Angione C. (2019). Machine and deep learning meet genome-scale metabolic modeling. PLoS Comput. Biol..

[bib79] Zhang S., Bryant D.A. (2011). The tricarboxylic acid cycle in cyanobacteria. Science.

