# Supplementary material for: A Hybrid Flux Balance Analysis and Machine Learning Pipeline Elucidates Metabolic Adaptation in Cyanobacteria
Source: iScience. 2020 Nov 18;23(12):101818. doi: 10.1016/j.isci.2020.101818 (PMC7744713; doi:10.1016/j.isci.2020.101818)
Supplement: Document S1. Transparent Methods, Figures S1–S4, and Tables S1–S13 [file mmc1.pdf]

iScience, Volume *23*

Supplemental Information

A Hybrid Flux Balance Analysis and Machine Learning Pipeline Elucidates Metabolic Adaptation in Cyanobacteria

Supreeta Vijayakumar, Pattanathu K.S.M. Rahman, and Claudio Angione

1. Transparent Methods

1.1. Growth Conditions

A complete list of the culture conditions for *Synechococcus* sp. PCC 7002 is reported in Table S1, for which RNA sequencing data was integrated into GSMM to generate a condition-specific FBA framework.

1.2. Condition-Specific Metabolic Modeling

We initiated our pipeline by mapping the gene expression profiles for phototrophic growth in *Synechococcus* sp. PCC 7002 using multi-omic flux balance analysis and building condition-specific flux profiles, starting from a model recently published by Hendry et al. (2016).

Transcriptomic data were acquired in the form of RNA-Seq data from a series of studies previously conducted by Ludwig and Bryant (Ludwig and Bryant, 2011, 2012a,b). Such data were compiled in an online repository known as CyanOmics (Yang et al., 2015), an integrated omics analysis database containing omic data specific to *Synechococcus* sp. PCC 7002. Specific growth conditions are recorded in Table S1, along with a reference to the original paper for each condition. For each culture condition, we downloaded the reads assigned per kilobase of target per million mapped reads (RPKMs) as a measure of relative transcript abundance (Yang et al., 2015). Given this information, we calculated fold change values of each gene dividing the RPKM values under these conditions by the average expression of three standard control replicates for that gene.

In flux balance analysis (FBA), a steady-state is assumed to calculate all fluxes under time-invariance and spatial homogeneity for purposes of mass conservation (Heirendt et al., 2019). Mass-balance constraints are imposed on the GSMM to identify a range of points representing all feasible flux distributions. A phenotypic state in the solution space is then computed using linear programming with a set of values indicating the optimal conditions required to optimize a given cellular objective function (Ebrahim et al., 2016).

In our pipeline, we integrated condition-specific expression profiles generated from RNA sequencing data with the *Synechococcus* GSMM using METRADE (Angione and Lió, 2015). We then implemented a quadratic program for solving a regularized bilevel FBA, using Gurobi as a quadratic programming solver.

The regularized optimization problem was formulated as:

$$\begin{aligned} \max \quad & g^{\intercal}v - \frac{\sigma}{2}\, v^{\intercal}v \\ \text{such that} \quad & \max f^{\intercal}v, \quad Sv = 0, \\ & v^{\min}\varphi(\Theta) \le v \le v^{\max}\varphi(\Theta), \end{aligned} \tag{1}$$

where S is the stoichiometric matrix recording all reactions and metabolites in the *Synechococcus* GSMM, v is the vector of reaction flux rates. f and g are Boolean vectors of weights selecting the reactions in v in which flux rates are considered as the objectives: f selects the primary objective function (biomass) and g selects the secondary objective function (ATP maintenance, photosystem I or photosystem II). In order to obtain a unique flux solution, the outer level objective g is regularized by subtracting the concave function $\frac{\sigma}{2}$ $v^{\intercal}v$, where $\sigma = 10^{-6}$ (Heirendt et al., 2019). Upon solving this problem, a vector of fluxes (v) is obtained.

$v^{\min}$ and $v^{\max}$ are vectors representing the lower- and upper-limits for the flux rates in v for the unconstrained model. The gene set expression of reactions associated with the fluxes in v are represented by the vector Θ. φ is a function mapping the expression level of each gene set to a coefficient for the lower- and upper-limits of the corresponding reaction (Angione et al., 2016), and is defined as follows:

$$\varphi(\Theta) = [\mathbf{1} + \gamma\,|log(\Theta)|]^{\mathrm{sgn}(\Theta - \mathbf{1})}. \tag{2}$$

In this way, a specific RNA-Seq profile was mapped onto the GSMM for each growth condition, and solving the quadratic optimization problem yielded condition-specific flux rates. In the case of standard control flux, the expression vector was set to all ones; for all other conditions, RNA-Seq data was mapped to coefficients for the lower and upper limits of the corresponding reactions using the function in Equation 2. γ represents the strength of gene expression mapped to each reaction in the model (set to 3.5). A sensitivity analysis was conducted to assess the robustness of this parameter, whilst ensuring maximum variability of experimentally-feasible flux values across growth conditions. Adjusting $\gamma = 3.5 \pm 0.1$ resulted in a flux change of approximately $\pm$ 0.0078 (averaged across all conditions).

1.3. Flux Constraints

The uptake rate (i.e. lower bound) of carbon dioxide was fixed at -10 under all growth conditions, except for the low CO_2 growth condition (-0.01). In order to establish a protocol for specifying the variation in light uptake across growth conditions, a photon uptake rate (P_U) was calculated for each growth condition using a method similar to Vu *et al.* (Vu et al., 2012). The available light consumption (LC) under each condition (mmol) was multiplied by the surface area (SA) of the culture exposed to the light source (m^2), then the product was divided by the total available dry cell weight (DCW) of the culture (g per volume):

$$P_U = \frac{LC \times SA}{DCW} \tag{3}$$

ID	Condition	Specifics	Ref.
1	Dark oxic	Incubated in darkness prior to harvest, sparged in N_2	(Ludwig and Bryant, 2011)
2	Dark anoxic	Incubated in darkness prior to harvest	(Ludwig and Bryant, 2011)
3	High light	Illuminated at 900 μmol photons m^{-2} s^{-1} prior to harvest	(Ludwig and Bryant, 2011)
4	OD 0.4	Harvested at OD 730nm = 0.4	(Ludwig and Bryant, 2011)
5	OD 1.0	Harvested at OD 730nm = 1.0	(Ludwig and Bryant, 2011)
6	OD 3.0	Harvested at OD 730nm = 3.0	(Ludwig and Bryant, 2011)
7	OD 5.0	Harvested at OD 730nm = 5.0	(Ludwig and Bryant, 2011)
8	Low O_2	Sparged in N_2	(Ludwig and Bryant, 2011)
9	Low CO_2	Sparged with air [0.035% (v/v) CO_2]	(Ludwig and Bryant, 2012a)
10	N-limited	Cells washed in medium A (lacking NO_3^-) and resuspended	(Ludwig and Bryant, 2012a)
11	S-limited	Cells washed with $MgCl_2$	(Ludwig and Bryant, 2012a)
12	PO_4^{3-} limited	Cells washed w/o (PO_4^{3-}) harvested at OD = 0.7	(Ludwig and Bryant, 2012a)
13	Fe-limited	Cells washed in medium A with 720 μM deferoxamine mesylate B added at OD 0.35	(Ludwig and Bryant, 2012a)
14	NO_3^-	Standard growth in medium A (lacking $NaNO_3$) with 25 mM HEPES, 1 μM NiSO4, 12 mM $NaNO_3$	(Ludwig and Bryant, 2012a)
15	NH_3	Standard growth in medium A (lacking $NaNO_3$) with 25 mM HEPES, 1 μM $NiSO_4$ and 10 mM NH_4Cl	(Ludwig and Bryant, 2012a)
16	$CO(NH_2)_2$	Standard growth in medium A (lacking $NaNO_3$) with 25 mM HEPES, 1 μM $NiSO_4$ and 10 mM $CO(NH_2)_2$	(Ludwig and Bryant, 2012a)
17	Heat Shock	1h heat shock at 47°C	(Ludwig and Bryant, 2012b)
18	22°C	Standard growth at 22°C	(Ludwig and Bryant, 2012b)
19	30°C	Standard growth at 30°C	(Ludwig and Bryant, 2012b)
20	Oxidative stress	5 μM methyl viologen added 30 minutes prior to harvesting	(Ludwig and Bryant, 2012b)
21	Mixotrophic	Medium A+ supplemented with 10 mM glycerol	(Ludwig and Bryant, 2012b)
22	Low salt	Medium A+ containing 3 mM NaCl and 0.08 mM KCl	(Ludwig and Bryant, 2012b)
23	High salt	Medium A+ containing 1.5 M NaCl and 40 mM KCl	(Ludwig and Bryant, 2012b)
24	Standard control	Medium A+ at 38°C (*see caption for full details*)	(Ludwig and Bryant, 2012b)

Table S1: **Growth and stress conditions for *Synechococcus* sp. PCC 7002, Related to Figure 2.** The standard control condition was defined as: Medium A+ at 38°C, illuminated at 250 μmol photons $m^{-2}s^{-1}$, sparged in air with 1% (v/v) CO_2, with cells harvested at OD 730nm = 0.7.

As per the culture conditions described by Ludwig and Bryant (Ludwig and Bryant, 2011), the surface area of the culture exposed to the light source was calculated using the diameter of the culture tube (20mm) and the volume of the culture medium (25ml). The DCW of marine *Synechococcus* has been approximated at 0.35 gDW/L for an optical density (OD) of 1.0 at 750nm (Myers et al., 2013), which is close to other DCW estimates for *Synechococci* (Aikawa et al., 2014; Qiao et al., 2018). Given this estimate, a pre-established linear calibration for *Synechococcus* PCC 7942 cultures was used to calculate DCW for optical densities above OD 1.8 (i.e. OD 3.0 and 5.0) (Kato et al., 2017). In the plot (y = DCW, x = OD), since the same line could not be extrapolated below OD = 1.8 due to yielding negative DCW values, we adopted a piecewise linear approximation, and a separate linear equation was calculated for OD 0.4 and 0.7 between the lowest OD point (1.8,0.375) and the origin (0,0). The full specification of constraints for each growth condition and further information on their calculation is available in Supplementary Data 3.

1.4. Data Normalization

When creating the combined transcript-and-flux dataset, the two types of data (gene transcripts and reaction fluxes) were combined by converting them into fold change values. The fold changes of the RNA-Seq data was obtained by dividing the RPKM values for each growth condition by the average expression of three standard control replicates for that gene, as detailed in Section 1.2. In order to scale flux rates in the same range as the gene transcripts, we converted flux rates into fold change values by dividing the flux rates under these conditions by the flux rate derived for the standard condition. This provided a ratio of reaction activity between experimental conditions and the standard control. In order to account for the FBA solver tolerance, all the negligible flux rates (rate < 10^{-4}) were set to zero. When both experimental and control flux rates were negligible, fold change values were set to 1, while fold changes for divisions resulting in infinite values (due to the control flux being zero) were set to the maximum fold change for that dataset.

1.5. Principal Component Analysis (PCA)

Ascertaining the contribution of each condition to the construction of each dimension allowed us to detect conditions that deviated from the usual patterns, as well as those that were the greatest contributors to variance in the dataset. The PCA was conducted using the FactoMineR package in R (Lê et al., 2008), where conditions were described by gene transcripts and/or reaction fluxes (quantitative variables) for each pair of objectives, and the contribution of each condition to the dimensions and variance in the datasets was recorded.

Figure 2(a-c) displays PCA individual factor maps for the three objective pairs, showing the principal component scores of 24 individuals (which in our case are simulated growth conditions) described by 742 fluxes on the first two principal components. Figure 3(a), (c), (e) and (g) display individual factor maps for the three objective pairs and the respective principal component scores of 24 individuals described by 3187 transcripts (a), or 3929 multi-omic variables (c), (e) and (g), which include both transcripts and fluxes.

1.6. K-means Clustering

Gene transcripts, flux rates, or a combination of both measurements were used as variables for clustering growth conditions. To specify the appropriate number of clusters for running the k-means algorithm, we performed a silhouette analysis to assess the accuracy of cluster assignment by measuring the cohesion of data points within each cluster (given by a silhouette value for each variable). Both silhouette analysis and clustering were performed in MATLAB with the *silhouette* and *kmeans* functions, using the number of clusters that returned the highest silhouette values for the majority of points ($k = 6$).

Whilst computing correlation between genes across the profiles, the *zscore* function was used to standardize each of the profiles to have zero mean and unit variance. The pattern of clustering using the "cityblock" distance metric was compared between: (i) transcripts only (ii), fluxes only, (iii) both gene transcripts and fluxes. Multidimensional scaling was performed using a robust variation of the *mdscale* function in Matlab to circumvent colocation of points by multiplying dissimilarities by a scalar value (minimizing the squared stress criterion with 500 iterations of the iterative algorithm). The results of this clustering are reported in Figure 2(d-f) and Figure 3(b), (d), (f) and (h).

1.7. LASSO Regression

To relate the calculated flux distributions to *in-vivo* growth in our model, x is the data matrix consisting of input variables in the form of (i) gene transcripts (ii) flux rates, or (iii) both gene transcripts and flux rates, and y is the vector of growth rates in Table S2. Of the 23 transcriptomic profiles procured from the original studies, only 12 growth conditions had available (i) specified growth rates, (ii) specified doubling times, or (iii) standard growth curves (Ludwig and Bryant, 2011, 2012a,b). For these growth curves, the gradient between OD 0.4 and OD 0.7 was calculated as the growth rate, and all the other growth rates and doubling times were calculated relative to the standard growth rate. Although growing photoautotrophically in nature, heterotrophically-grown microalgae sometimes have higher growth rates, in the absence

of light shading and more space available to achieve greater cellular density (Kim et al., 2016).

Condition	Doubling time	Growth rate
Standard	100.000	0.075
N-limited	162.500	0.046
S-limited	150.000	0.050
P-limited	212.500	0.035
Nitrate	43.281	0.173
Ammonia	28.133	0.267
Urea	28.133	0.267
22°C	194.000	0.039
30°C	109.000	0.069
Mixotrophic	84.000	0.089
Low salt	98.000	0.077
High salt	270.000	0.028

Table S2: **Growth rates for LASSO and correlation analysis, Related to Figures 5 and 6.** Growth rates of 12 conditions calculated from growth curves previously published for *Synechococcus* sp. PCC 7002 (Ludwig and Bryant, 2011, 2012a,b). Doubling time is given as a fraction of standard conditions (where 100 is the standard condition).

The formula for the LASSO algorithm is specified in Eq. 4. We solved the regression problem in the form:

$$\min_{\beta_0,\beta}\left(\frac{1}{2N}\sum_{i=1}^{N}(y_i-\beta_0-x_i^T\beta)^2+\lambda\sum_{j=1}^{p}|\beta_j|\right), \quad (4)$$

where N is the number of conditions, y_i is the growth rate at the (i)th condition, p is the number of predictors indexed by j, x_i is a vector of p values at condition i, λ is a positive regularization parameter and β_0 and β represent the scalar and p-vector coefficients.

Our LASSO analysis reduced the list of recursive features by retaining positive and negative non-zero coefficients greater than 0.01. Furthermore, to provide a measure of predictive accuracy, we calculated the mean squared error (MSE) for all the fitted coefficients yielded as a result of the LASSO regression in each dataset. These values are recorded in Supplementary Data 2. The errors were at least one magnitude below the observed growth rates (in the response variable y), showing low deviation/variance between predicted and actual values and therefore good predictive accuracy overall.

1.8. Correlation Analysis

To find the strength of the association between growth rates and gene expression or flux values, the Pearson correlation coefficient was calculated between each vector of gene transcripts/flux rates and growth rates across conditions (see Table S2). For this analysis, all flux fold changes were converted into absolute (non-negative) values before calculating the correlation, therefore considering reversible reaction fluxes in absolute value to represent the activity of that reaction. For each gene/reaction, the mean predictor coefficient (MPC) was calculated by averaging across coefficients in all vectors for that predictor. The highest positively/negatively correlated genes or reactions for each dataset are plotted in Figure 5.

2. Supplemental Results

2.1. Clustering

For Biomass-ATP maintenance fluxes (Figure 2d), the clusters formed were: (i) dark oxic, dark anoxic, low O_2, low CO_2, nitrogen limitation, sulfur limitation, nitrate, ammonia, heat shock and mixotrophic; (ii) high light intensity, phosphate limitation, and standard control; (iii) OD 0.4, OD 3.0, 30°C and oxidative stress; (iv) OD 1.0 and urea; (v) OD 5.0, iron limitation and high salinity; (vi) 22°C and low salinity.

For Biomass-Photosystem I fluxes (Figure 2e), the clusters were: (i) dark oxic, dark anoxic, OD 3.0, OD 5.0, phosphate limitation, heat shock, mixotrophic and low salinity; (ii) high light intensity; (iii) OD 0.4; (iv) OD 1.0, nitrogen limitation and sulfur limitation; (v) low O_2 (vi) low CO_2, iron limitation, nitrate, ammonia, urea, 22°C, 30°C, oxidative stress, high salinity, and standard control.

For Biomass-Photosystem II fluxes (Figure 2f), the clusters were: (i) dark oxic, dark anoxic, OD 3.0, OD 5.0, phosphate limitation, heat shock, mixotrophic and low salinity; (ii) high light intensity; (iii) OD 0.4; (iv) OD 1.0, nitrogen limitation and sulfur limitation; (v) low O_2 (vi) low CO_2, iron limitation, nitrate, ammonia, urea, 22°C, 30°C, oxidative stress, high salinity, and standard control.

For the gene transcripts (plots a-b in Figure 3) the clusters formed were: (i) dark oxic, high light intensity, OD 0.4, OD 1.0, OD 3.0, OD 5.0, low O_2, nitrogen limitation, sulfur limitation, phosphate limitation, nitrate, ammonia, urea and low salinity; (ii) dark anoxic; (iii) low CO_2; (iv) iron limitation; (v) heat shock; (vi) 22°C, 30°C, oxidative stress, high salinity, and standard control.

When combining both transcript and flux data (Figure 3 c-h), the clusters formed were less distinct. For the Biomass-ATP objective pair, the clusters were: (i) dark oxic, OD 1.0, OD 3.0, low CO_2, nitrogen limitation, sulfur limitation, iron limitation, nitrate, ammonia, urea, 30°C, oxidative stress and high salinity; (ii) dark anoxic, low O_2, phosphate limitation, heat shock, mixotrophic, and standard control; (iii) high light intensity; (iv) OD 0.4 (v) OD 5.0 (vi) 22°C and low salinity.

For the Biomass-PI objective pair, the clusters were: (i) dark oxic, OD 1.0, OD 3.0, OD 5.0, low O_2, low CO_2, nitrogen limitation, sulfur limitation, nitrate,

ammonia, urea, heat shock, 22°C, 30°C, oxidative stress, high salinity, and standard control; (ii) dark anoxic; (iii) high light intensity and OD 0.4; (iv) phosphate limitation and low salinity; (v) iron limitation; (vi) mixotrophic.

For the Biomass-PII objective pair, the clusters were: (i) dark oxic, OD 1.0, OD 3.0, OD 5.0, low O_2, low CO_2, nitrogen limitation, iron limitation, nitrate, ammonia, urea, heat shock, 22°C, 30°C, oxidative stress, high salinity, and standard control; (ii) dark anoxic; (iii) high light intensity and OD 0.4; (iv) sulfur limitation; (v) phosphate limitation; (vi) mixotrophic and low salinity.

2.2. Principal Component Contributions to Variance

Table S3 displays contributions of the fluxes to the principal components in the form of a list of the top ten reactions contributing to variation in the datasets for all three objective pairs. Among the highest contributors to variance for all objectives was inorganic diphosphate (IODP), which catalyzes the hydrolysis of phosphorous acid anhydrides. Additionally, aspartate transaminase (ASPTA1) is involved in the transfer of an amino group from aspartate to alpha-ketoglutarate during which glutamate and oxaloacetate are formed. In the absence of sufficient oxygen, photoautotrophs such as *Synechococcus* are capable of switching from aerobic respiration to anaerobic fermentation in order to produce ATP for cellular metabolic processes. For all objective pairs, formate exchange (EX_FOR_E) and transport (FORT) were among the highest contributors to variance. Methenyltetrahydrofolate cyclohydrolase (MTHFC) and methylenetetrahydrofolate dehydrogenase (MTHFD) are intermediates in folate biosynthesis that are involved in the Wood-Ljungdahl pathway (aka reductive acetyl coenzyme A pathway), where carbon fixation by acetyl-CoA synthase and fermentative respiration occur concurrently and acetate is generated as the end product((Woo and Jang, 2019)). The exchange and transport of formate (EX_FOR_E and FORT) are also linked with folate biosynthesis, since formate can be converted into tetrahydrofolate. Transketolase (TK1 and TK2) as well as sedoheptulose bisphosphatase are also utilized in the pentose phosphate pathway. The pentose phosphate pathway is an important source of NADPH, a reducing agent used to drive the numerous oxidation-reduction reactions throughout the central carbon metabolic pathways. In *Synechocystis* sp. PCC 6803, higher metabolic flux was observed for NADPH production under low light conditions since the oxidative pentose phosphate pathway provides an alternative route for NADPH production (Ueda et al., 2018). For the biomass-photosystem I objective pair, glutamate exchange (EX_GLU_E) and transport of L-glutamate(GLUSYM) and sodium (NAT3) were represented in the highest contributors. For the biomass-photosystem II objective pair, the key respiratory enzymes glucose-6-isomerase (PGI) and pyruvate dehydrogenase (PDH) as well as ribulose 5-phosphate 3-epimerase (RPE) from the pentose phosphate pathway were included. The pentose phosphate pathway runs parallel to glycolysis and is mainly responsible for the synthesis of amino acid and nucleotide precursors. Pyruvate dehydrogenase is among the most important enzymes in central carbon metabolism since it is responsible for producing acetyl-CoA.

2.3. LASSO Regression

Reducing the number of predictors in a regression model enables identification of important predictors, elimination of redundant predictions, and generation of shrinkage estimates with lower predictive errors than ordinary least squares regression. LASSO employs L1 regularization, which penalizes the sum of absolute values of all the coefficients; this sets the coefficients of unnecessary or recursive features equal to zero, resulting in a sparser matrix. The formula for the algorithm is specified in Eq. 4 in the main text. Tables S6, S7, and 2.3 list all non-zero coefficients and the transcripts/fluxes they are associated with. For each gene/reaction, the mean predictor coefficient (MPC) is calculated by averaging across coefficients in all vectors for that predictor.

When applying the LASSO algorithm to the flux-only dataset (Table S7), the coefficients identified primarily belonged to pathways associated with the metabolism of nucleotides, co-factors and vitamins, but also those that converge in order to fulfil a common objective i.e. energy generation from carbohydrate and lipid metabolism, or the exchange and transport of specific metal ions. Of particular interest is cobalt transport (COBALTT5), since this reaction yielded one of the highest coefficient values. Many cyanobacterial species (including *Synechococcus*) utilize nickel, copper, zinc, and cobalt-containing enzymes as protein co-factors that constitute important components of the photosynthetic machinery (Huertas et al., 2014). Furthermore, these co-factors contribute to mechanisms for survival in iron-depleted environments (Palenik et al., 2003), such as the secretion of siderophores to chelate iron. In the form of the photosystem II manganese-stabilizing polypeptide (psbO), manganese plays a critical role in photosystem II function by catalyzing the light-induced dissociation of water to molecular oxygen (Bartsevich and Pakrasi, 1995; Liu et al., 2018). Inactivation of the manganese transport system (MNABC) leads to an invariable loss of activity in photosystem II, consequently affecting the photosynthetic process as a whole (Shcolnick and Keren, 2006). Similarly, the molybdenum exchange and transport reactions (EX_MOBD_E and MOBDABC) represent the

**Biomass - ATP maintenance**				
No.	**Rxn**	Reaction Name	Dim 1	Dim 2
1	IODP	inorganic diphosphatase	0.307	0.012
2	TKT1	transketolase	0.307	0.016
3	NDPK1	nucleoside-diphosphate kinase (ATP:GDP)	0.307	0.016
4	RPE	ribulose 5-phosphate 3-epimerase	0.307	0.016
5	TKT2	transketolase	0.307	0.016
6	FBA3	Sedoheptulose 1,7-bisphosphate D-glyceraldehyde-3-phosphate-lyase	0.307	0.016
7	SBP	sedoheptulose-bisphosphatase	0.307	0.016
8	FBA	fructose-bisphosphate aldolase	0.307	0.016
9	FBP	fructose-bisphosphatase	0.307	0.016
10	NDPK3	nucleoside-diphosphate kinase (ATP:CDP)	0.307	0.016
**Biomass - Photosystem I**				
No.	**Rxn**	Reaction Name	Dim 1	Dim 2
1	ASPTA1	aspartate transaminase	0.270	0.001
2	GLUSYM	L-Glutamate transport in via sodium symport	0.270	0.004
3	EX_GLU_E	glutamate exchange	0.270	0.004
4	NAT3	sodium transport out via proton antiport	0.270	0.004
5	IODP	inorganic diphosphatase	0.270	0.004
6	EX_FOR_E	formate exchange	0.270	0.003
7	FORT	formate transport via diffusion	0.269	0.003
8	MTHFC	methenyltetrahydrofolate cyclohydrolase	0.269	0.004
9	MTHFD	methylenetetrahydrofolate dehydrogenase (NADP)	0.269	0.004
10	FTHFD	formyltetrahydrofolate deformylase	0.269	0.003
**Biomass - Photosystem II**				
No.	**Rxn**	Reaction Name	Dim 1	Dim 2
1	ASPTA1	aspartate transaminase	0.272	1.36×10^{-5}
2	IODP	inorganic diphosphatase	0.272	0.001
3	PDH	pyruvate dehydrogenase	0.272	0.001
4	MTHFC	methenyltetrahydrofolate cyclohydrolase	0.272	4.49×10^{-4}
5	MTHFD	methylenetetrahydrofolate dehydrogenase (NADP)	0.272	4.49×10^{-4}
6	TKT1	transketolase	0.272	3.57×10^{-4}
7	EX_FOR_E	formate exchange	0.272	1.23×10^{-4}
8	FORT	formate transport via diffusion	0.272	0.001
9	PGI	glucose-6-phosphate isomerase	0.272	0.001
10	RPE	ribulose 5-phosphate 3-epimerase	0.272	0.001

Table S3: **Contributions of reactions to variance, Related to Figure 2.** Top ten contributions of individual reaction fluxes to principal component variables for the first two dimensions when PCA is performed on the fluxes for three pairs of objectives (biomass-ATP maintenance, biomass-photosystem I, biomass-photosystem II).

**No.**	**Gene**	**COG category**	**CY category**	**CY subcategory**	**Dim 1**	**Dim 2**
1	A0445	AA transport and metabolism	Transport and binding proteins	NA	0.125	0.002
2	A1216	Translation, ribosomal structure and biogenesis	Translation	Aminoacyl tRNA synthetases and tRNA modification	0.124	5.16×10^{-5}
3	A0327	Energy production and conversion	Hypothetical	NA	0.124	0.007
4	A1341	None	Unknown	NA	0.123	0.0006
5	A1274	AA transport and metabolism/Cell wall, membrane, envelope biogenesis	AA biosynthesis	Aspartate family	0.121	0.0008
6	A0831	Coenzyme transport and metabolism	Biosynthesis of cofactors, prosthetic groups, and carriers	Thiamin	0.119	8.66×10^{-5}
7	A1173	Secondary metabolites biosynthesis, transport and metabolism	Fatty acid, phospholipid and sterol metabolism	NA	0.119	0.0003
8	A2554	Carbohydrate transport and metabolism	Transport and binding proteins	NA	0.111	7.59×10^{-8}
9	A0939	Signal transduction mechanisms	Hypothetical	NA	0.111	0.011
10	A1414	Energy production and conversion	Energy Metabolism	Pyruvate and acetyl-CoA metabolism	0.110	0.002

Table S4: **Contributions of genes to variance, Related to Figure 3.** Top ten contributions of genes to principal component variables for the first two dimensions when PCA is performed on the gene transcripts. COG category refers to the Cluster of Orthologous Groups that the gene belongs to, whilst CY category is its functional category according to Cyanobase (Fujisawa et al., 2016).

No.	Gene/Rxn	COG category/Rxn description	CY category/subsystem	CY subcategory	Dim 1	Dim 2
**Biomass - ATP maintenance**						
1	A0831	Coenzyme transport and metabolism	Biosynthesis of cofactors, prosthetic groups, and carriers	Thiamin	0.118	3.31×10^{-5}
2	A1274	AA transport and metabolism/Cell wall, membrane, envelope biogenesis	Amino acid biosynthesis	Aspartate family	0.118	0.002
3	A1216	Translation, ribosomal structure and biogenesis	Translation	Aminoacyl tRNA synthetases and tRNA modification	0.118	0.002
4	A0445	AA transport and metabolism	Transport and binding proteins	NA	0.117	0.002
5	A1341	None	Unknown	NA	0.116	0.005
6	A0327	Energy production and conversion	Hypothetical	NA	0.115	0.012
7	A1173	Secondary metabolites biosynthesis, transport and metabolism	Fatty acid, phospholipid and sterol metabolism	NA	0.115	0.003
8	A2796	Replication, recombination, and repair	DNA replication, restriction, modification, recombination, and repair	NA	0.110	0.003
9	A0743	Lipid transport and metabolism	Other	Other	0.107	0.003
10	A1414	Energy production and conversion	Energy Metabolism	Pyruvate and acetyl-CoA metabolism	0.107	0.003

No.	Gene/Rxn	COG category/Rxn description	CY category/subsystem	CY subcategory	Dim 1	Dim 2
**Biomass - Photosystem I**						
1	A0831	Coenzyme transport and metabolism	Biosynthesis of cofactors, prosthetic groups, and carriers	Thiamin	0.119	0.001
2	A1274	AA transport and metabolism/Cell wall, membrane, envelope biogenesis	Amino acid biosynthesis	Aspartate family	0.115	0.005
3	A0445	AA transport and metabolism	Transport and binding proteins	NA	0.114	0.006
4	A1216	Translation, ribosomal structure and biogenesis	Translation	Aminoacyl tRNA synthetases and tRNA modification	0.114	0.007
5	A1341	None	Unknown	NA	0.112	0.009
6	A1173	Secondary metabolites biosynthesis, transport and metabolism	Fatty acid, phospholipid and sterol metabolism	NA	0.111	0.005
7	A0327	Energy production and conversion	Hypothetical	NA	0.111	0.016
8	A2796	Replication, recombination, and repair	DNA replication, restriction, modification, recombination, and repair	NA	0.110	0.001
9	A0743	Lipid transport and metabolism	Other	Others	0.107	2.69×10^{-4}
10	A1405	Signal transduction mechanisms	Other	Drug and analog sensitivity	0.105	0.001

No.	Gene/Rxn	COG category/Rxn description	CY category/subsystem	CY subcategory	Dim 1	Dim 2
**Biomass - Photosystem II**						
1	A0831	Coenzyme transport and metabolism	Biosynthesis of cofactors, prosthetic groups, and carriers	Thiamin	0.119	7.48×10^{-4}
2	A1274	AA transport and metabolism/Cell wall, membrane, envelope biogenesis	Amino acid biosynthesis	Aspartate family	0.115	0.005
3	A0445	AA transport and metabolism	Transport and binding proteins	NA	0.115	0.006
4	A1216	Translation, ribosomal structure and biogenesis	Translation	Aminoacyl tRNA synthetases and tRNA modification	0.114	0.007
5	A1341	None	Unknown	NA	0.113	0.009
6	A1173	Secondary metabolites biosynthesis, transport and metabolism	Fatty acid, phospholipid and sterol metabolism	NA	0.112	0.005
7	A0327	Energy production and conversion	Hypothetical	NA	0.111	0.016
8	A2796	Replication, recombination, and repair	DNA replication, restriction, modification, recombination, and repair	NA	0.111	0.001
9	A0743	Lipid transport and metabolism	Other	Other	0.108	2.57×10^{-4}
10	A1405	Signal transduction mechanisms	Other	Drug and analog sensitivity	0.106	0.001

Table S5: **Contributions of genes and reactions to variance, Related to Figure 3.** Top ten contributions of gene transcripts and reaction fluxes to principal component variables for the first two dimensions when PCA is performed on the gene transcripts and fluxes for three pairs of objectives (biomass-ATP maintenance, biomass-photosystem I, biomass-photosystem II).

uptake and transport of molybdenum by cyanobacterial ABC transporters to form cofactors (e.g. Moco or FeMoco) that assist in nitrogen fixation (Shvarev and Maldener, 2018; Demtröder et al., 2019). GMPS2 (guanosine monophosphate synthetase) was among the top negative LASSO coefficients and catalyzes the reversible conversion between xanthosine 5' phosphate and GMP (guanosine monophosphate) and also between L-glutamine and L-glutamate. Significantly, the nitrate reduction reaction (FDNOR1) was one of the main negative LASSO coefficients retained in the Biomass-Photosystem I flux data. Ferredoxins are iron and sulfur-containing proteins that act as electron carriers during many important metabolic processes including oxidation-reduction, photosynthesis, and nitrogen fixation (Lea-Smith et al., 2016). More specifically, nitrate ferredoxin acts as a significant electron sink during the reversible, redox inter-conversion of nitrite to nitrate during photosynthesis (Flores et al., 2005; Qian et al., 2016).

The positive coefficients for reactions previously identified during PCA were also retained for the Biomass-Photosystem I objective pair. ASPO5 (anaerobic L-aspartate oxidase) was identified as the highest positive LASSO coefficient in the ATP objective pair. This anaerobic reaction uses fumarate as a terminal electron acceptor instead of oxygen, producing succinate.

Succinate dehydrogenase (SUCD1Itlm and SUCD1Icpm) catalyzes the interconversion of succinate to fumarate as a product of NADH oxidation in the TCA cycle, a process which can still occur in *Synechococcus* sp. PCC 7002 during fermentative metabolism in dark, anoxic conditions (McNeely et al., 2010). In a diurnal GSMM accounting for fluctuations in light availability in the phototrophic metabolism of *Synechocystis* sp. PCC 6803, an increased flux towards succinate was observed during the dark period, driving ATP production through respiratory electron transport (Sarkar et al., 2019). Other correlated reactions were associated with the oxidation, transport and exchange of D-lactate and water, synthesis of intermediates used in central carbon metabolism, and for synthesis of intermediates formed during amino acid biosynthesis and catabolism. The negatively correlated reactions included dehydrogenases in the TCA cycle that catalyze the reversible oxidation of carbohydrates or amino acids (L-aspartate, L-alanine, D-lactate) into pyruvate or oxaloacetate, which can be utilized in the central metabolic pathways (glycolysis or the TCA cycle).

2.4. Pearson Correlation Analysis

Similarly to LASSO, the Pearson correlation coefficient was calculated between each dataset of gene transcripts/flux rates (x) and growth rates (y) listed in Table S2 in the main text. For this analysis, all transcript and flux fold changes were converted into absolute (non-negative) values. Plots for the top ten positively/negatively correlated genes are given in Figure S1. Tables S9, S10, S11, S12, and 2.4 provide additional information on the function of each gene or reaction as well as its Pearson correlation coefficient. Additionally, Figure 6 (c) displays the mean absolute PCC values according to subsystems defined in the GSMM.

For all three objective pairs, genes involved in succinate dehydrogenation (SUCD1Itlm/SUCD1Icpm), efflux (SUCCt2b) or exchange (EX_succ_E) were found to be positively correlated with growth. In addition to succinate dehydrogenase, fumarase (FUMH), succinate semialdehyde dehydrogenase (SSALY) and 2-oxoglutarate (2OGDC) were positively correlated with growth in Biomass-ATP maintenance. All three of these enzymes play an important role in the interconversion of compounds within the TCA cycle. Overall, there were a high number of reactions that yielded similar positive coefficients for Biomass - Photosystem I and Biomass - Photosystem II data (Table 2.4). These reactions belonged to pathways relating to amino acid, carbohydrate, exchange and transport metabolism. NADPH dehydrogenases are among the top negative coefficients for all three flux datasets. Cyanobacteria consume a large amount of the cofactors NADPH and NADH whilst reducing nitrate to catabolize glycogen under dark anaerobic conditions (Qian et al., 2016). NADH dehydrogenase (type II) is a protein that catalyzes the electron transfer from NADH to a quinone molecule via a flavin co-factor (Heikal et al., 2014). In the case of *Synechococcus* and other cyanobacteria, this molecule is plastoquinone, which plays a critical role as a mobile electron carrier during the light-dependent reactions of photosynthesis. NADH type II is formed when plastoquinone combines with NADH to form NADH:plastoquinone oxidoreductase, which acts as a single-subunit flavoenzyme. In photosystem II, plastoquinone is doubly reduced to plastoquinol, acting as a terminal electron acceptor (McConnell et al., 2011). The reduced plastoquinone (plastiquinol) pool serves as an electron buffer, maintaining a consistent charge between photosystem II and photosystem I (Peltier et al., 2016). The reduction from plastiquinone to plastiquinol can occur both in the cytoplasm and the thylakoid membrane, either with or without a proton pump. Reactions for water exchange (EX_H20_E) and transport (H20T5) have the highest negative correlation with growth in the Biomass-ATP maintenance flux data. In addition to the photosystem II reaction (PSIIR), water and oxygen exchanged between the thylakoid lumen and cytosol are among the top negative coefficients in the Biomass-Photosystem II flux data. In cyanobacteria, water plays an important role in photo-

Gene	COG category	CY category	CY subcategory	MPC
A0639	None	Photosynthesis and respiration	Phycobilisome	0.036
A0575	None	Hypothetical	NA	0.012
A0720	Posttranslational modification, protein turnover, chaperones	Central intermediary metabolism	Others	0.011
A0083	General	Translation	Degradation of proteins peptides and glycopeptides	-0.012
A1376	Cell cycle control, cell division, chromosome partitioning	Hypothetical	NA	-0.025
G0060	Inorganic ion transport and metabolism	Transport and binding proteins	NA	-0.049

Table S6: **Non-zero coefficients (>0.01) retained by LASSO regularizer for all gene transcripts, Related to Section 3.4 LASSO regression and Figure 5.** The mean predictor coefficient (MPC) is calculated by averaging across coefficients in all vectors for that predictor. Genes yielding positive coefficients are associated with photosynthesis, respiration, and protein modification. Genes yielding negative predictor coefficients are involved in protein degradation, cell division, and transport of inorganic ions.

**Biomass - ATP maintenance**			
Reaction	Description	Subsystem	MPC
ASPO5	L-aspartate oxidase	AA Metabolism	11.253
GLNS	glutamine synthetase	AA Carbohydrate and Energy Metabolism	2.387
ILEABC	L-isoleucine transport via ABC system	Extracellular Transport	0.247
LEUABC	L-leucine transport via ABC system	Extracellular Transport	0.063
GMPS2	GMP synthase (glutamine-hydrolysing)	Nucleotide Metabolism	-3.589
IMPD	IMP dehydrogenase	Nucleotide Metabolism	-0.192
**Biomass - Photosystem I**			
Reaction	Description	Subsystem	MPC
COBALTT5	cobalt transport in/out via permease (no H+)	Transport	267.011
GLUSZ	glutamate synthase (Ferredoxin)	Energy and Carbohydrate Metabolism	88.057
ADSL2R	adenylosuccinate lyase	Nucleotide Metabolism and AA Metabolism	45.372
ATPM	ATP maintenance requirement	Nucleotide Metabolism	41.438
NTD7	5”-nucleotidase (AMP)	Nucleotide Metabolism	38.909
PRAGS	phosphoribosylglycinamide synthetase	Nucleotide Metabolism	14.157
ADCL	4-aminobenzoate synthase	Metabolism of cofactors and vitamins	13.206
THFAT	tetrahydrofolate aminomethyltransferase	Metabolism of cofactors and vitamins	12.862
PIABC	phosphate transport via ABC system	Transport	11.172
EX_GLYC_E	glycerol exchange	Exchange Reaction	9.735
ME2	malic enzyme (NADP)	Carbohydrate Metabolism and Energy Metabolism	-176.122
MNABC	manganese transport via ABC system	Transport	-3.832
FDNOR1	ferredoxin-NADP reductase	Energy Metabolism	-3.641
HTDHL6	(3R)-3-Hydroxypalmitoyl-[acyl-carrier-protein] hydro-lyase	Lipid metabolism and Metabolism of cofactors and vitamins	-2.140
HOXPRX	2-hydroxy-3-oxopropionate reductase (NAD)	Carbohydrate Metabolism	-1.509
BTMAT1	Butyryl-[acyl-carrier protein]:malonyl-CoA C-acyltransferase	Metabolism of cofactors and vitamins	-1.098
GTPCI	GTP cyclohydrolase I	Metabolism of cofactors and vitamins	-0.950
EX_COBALT2_E	cobalt exchange	Exchange Reaction	-0.913
DB4PS	3,4-Dihydroxy-2-butanone-4-phosphate	Metabolism of cofactors and vitamins	-0.821
GAPD_NADP	glyceraldehyde-3-phosphate dehydrogenase (NADP) (phosphorylating)	Energy Metabolism	-0.817
**Biomass - Photosystem II**			
Reaction	Description	Subsystem	MPC
ADCL	4-aminobenzoate synthase	Metabolism of cofactors and vitamins	367.805
EX_GLYC_E	glycerol exchange	Exchange Reaction	119.944
G3PD2	glycerol-3-phosphate dehydrogenase (NADP)	Lipid metabolism	61.787
ALALIG	D-alanine-D-alanine ligase (reversible)	Cell wall and Metabolism of other amino acids	48.533
EX_MN2_E	manganese exchange	Exchange Reaction	4.757
EX_PTRC_E	putrescine exchange	Exchange Reaction	4.011
PTRCABC	putrescine transport via ABC system	Extracellular Transport	0.301
GMPS2	GMP synthase (glutamine-hydrolysing)	Nucleotide Metabolism	-0.829

Table S7: **Non-zero coefficients (>0.01) retained by LASSO regularizer for fluxes in the three pairs of objectives, Related to Section 3.4 LASSO regression and Figure 5.** Flux rates below 10^{-4} were not considered in order to account for solver tolerance during optimization. The coefficients retained are related to pathways involved in energy metabolism - fatty acid synthesis, transport/exchange, nucleotide metabolism, carbohydrate metabolism, and amino-acid metabolism.

**Biomass - ATP maintenance**				
Gene/Rxn	COG category/Rxn description	CY category/subsystem	CY subcategory	MPC
A0639	None	Photosynthesis and respiration	Phycobilisome	0.036
A0575	None	Hypothetical	NA	0.012
A0720	Posttranslational modification, protein turnover, chaperones	Central intermediary metabolism	Others	0.011
G0060	Inorganic ion transport and metabolism	Transport and binding proteins	NA	-0.049
A1376	Cell cycle control, cell division, chromosome partitioning	Hypothetical	NA	-0.025
A0083	General	Translation	Degradation of proteins peptides and glycopeptides	-0.012
**Biomass - Photosystem I**				
Gene/Rxn	COG category/Rxn description	CY category/subsystem	CY subcategory	MPC
A0639	None	Photosynthesis and respiration	Phycobilisome	0.036
A0575	None	Hypothetical	NA	0.012
A0720	Posttranslational modification, protein turnover, chaperones	Central intermediary metabolism	Others	0.011
G0060	Inorganic ion transport and metabolism	Transport and binding proteins	NA	-0.049
A1376	Cell cycle control, cell division, chromosome partitioning	Hypothetical	NA	-0.025
A0083	General	Translation	Degradation of proteins peptides and glycopeptides	-0.012
**Biomass - Photosystem II**				
Gene/Rxn	COG category/Rxn description	CY category/subsystem	CY subcategory	MPC
A0639	None	Photosynthesis and respiration	Phycobilisome	0.036
A0575	None	Hypothetical	NA	0.012
A0720	Posttranslational modification, protein turnover, chaperones	Central intermediary metabolism	Others	0.011
G0060	Inorganic ion transport and metabolism	Transport and binding proteins	NA	-0.049
A1376	Cell cycle control, cell division, chromosome partitioning	Hypothetical	NA	-0.025
A0083	General	Translation	Degradation of proteins peptides and glycopeptides	-0.012

Table S8: **Non-zero coefficients (>0.01) retained by LASSO regularizer for all features (gene transcripts and fluxes) with the three objective pairs, Related to Section 3.4 LASSO regression and Figure 5.** Only gene coefficients are retained, which are the same predictors as Table S6.

system II when it splits to produce molecular oxygen during the light-driven flux of protons from the cytoplasm to the luminal side of the thylakoid membrane, enabling a transmembrane electric potential for the diffusion of metal ions (Checchetto et al., 2012). Similar to results of the LASSO, the nitrate reduction reaction (FDNOR1) has the highest negative Pearson coefficient in the Biomass-Photosystem I flux data. The NAD-dependent malic enzyme (ME2) yielded a highly positive correlation coefficient for the Biomass- ATP maintenance data but a negative coefficient for the Biomass - Photosystem I data. Its function within the TCA cycle is the oxidative decarboxylation of malate and NAD+ to pyruvate and NADH.

When the gene transcripts were analyzed in isolation (Table S9), the highest Pearson correlation coefficients were given by (i) genes relating to photosynthesis and energy metabolism (A0639, A1008, A1802), and (ii) genes relating to amino acid biosynthesis and protein assembly (A2457, A0881). The phycobilisome is a large, light-harvesting membrane complex in photosystem II comprising chromophorylated phycobiliproteins and linker peptides (Boulay et al., 2008). Phycobilisomes assist cyanobacteria in adapting to varying light conditions by adjusting their size and structure to accommodate the flow of energy (Noreña-Caro and Benton, 2018). These are evidently important features to retain in the model for a phototrophic cyanobacterium as photophysiological measurements associated with photosystems tend to increase with the growth rate (Watanabe et al., 2014). Correspondingly, nutrient limitation causes degradation of the phycobilisomes, which activates mechanisms responsible for maintaining photosynthetic efficiency (Jackson et al., 2015; Clark et al., 2018; Saha et al., 2016).

When the gene expression and flux datasets were combined, all of the same genes are identified in Table 2.4 as in Table S9 and there was no difference in coefficients between the objectives (i.e. Biomass - ATP maintenance, Biomass - Photosystem I, Biomass - Photosystem II). This supports our idea of using fluxes separately as additional predictive omic features in further machine learning analyses (Zampieri et al., 2019; Yang et al., 2019; Culley et al., 2020; Zhang et al., 2020), for predicting growth rate or production rates for biotechnologically-relevant metabolites.

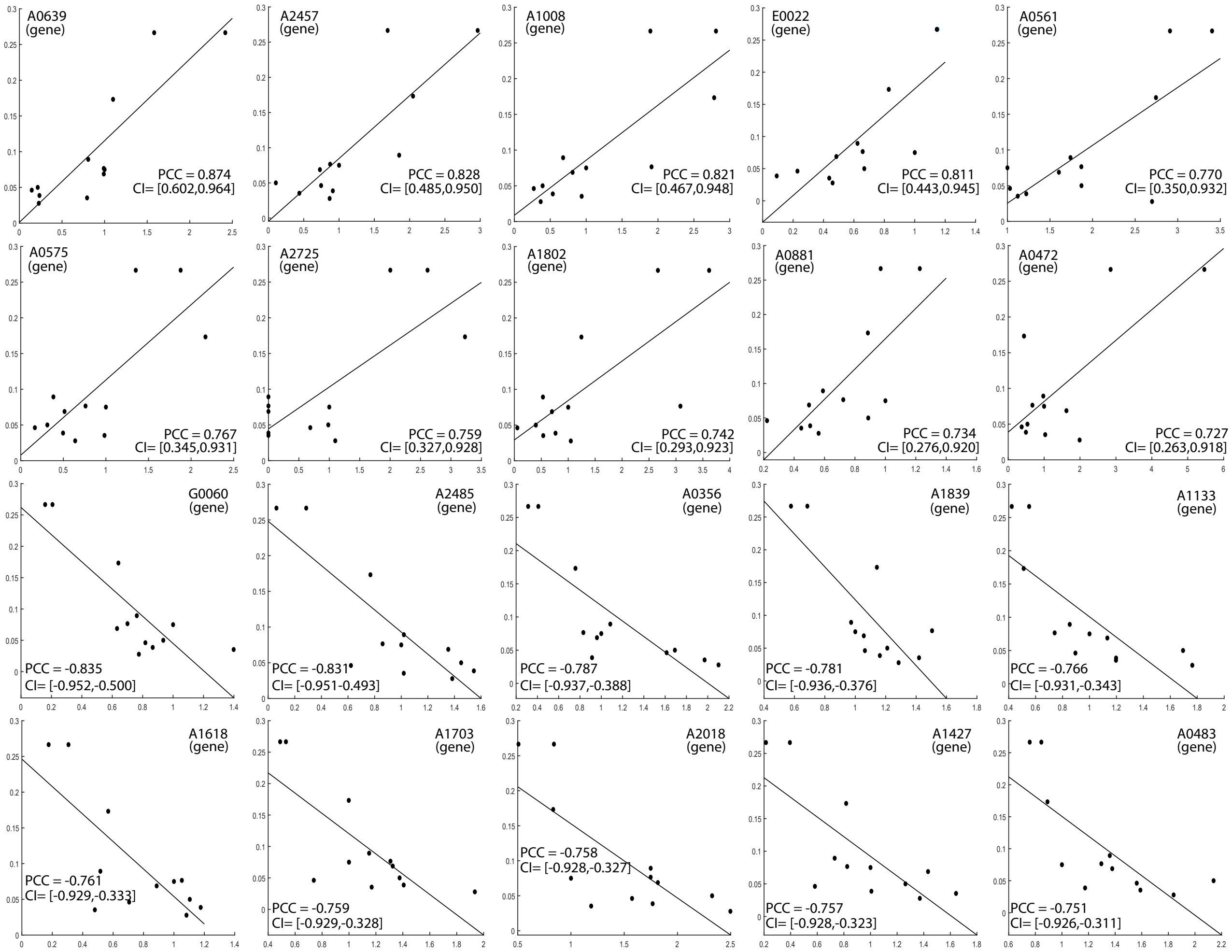

Figure S1: **Top ten Pearson correlation coefficients (PCC) with their respective 95% confidence intervals (CI) between gene transcript values (x) and growth rates (y), Related to Figure 5.** A list of each gene and its respective PCC is provided in Table S9.

Gene	COG category	CY category	CY subcategory	PCC
A0639	None	Photosynthesis and respiration	Phycobilisome	0.874
A2457	Post-translational modification, protein turnover, chaperones	Cellular processes	Chaperones	0.828
A1008	None	Photosynthesis and respiration	Photosystem I	0.821
E0022	None	Unknown	NA	0.811
A0561	None	Hypothetical	NA	0.770
A0575	None	Hypothetical	NA	0.767
A2725	None	NA	NA	0.759
A1802	Energy production and conversion/Secondary metabolites biosynthesis, transport and metabolism	Photosynthesis and respiration	CO_2 fixation	0.742
A0881	AA transport and metabolism	AA biosynthesis	Aspartate family	0.734
A0472	None	Hypothetical	NA	0.727
G0060	Inorganic ion transport and metabolism	Transport and binding proteins	NA	-0.835
A2485	General	Hypothetical	NA	-0.831
A0356	General	Translation	Degradation of proteins peptides and glycopeptides	-0.787
A1839	None	Other	Other	-0.781
A1133	Replication, recombination, and repair	Translation	Aminoacyl tRNA synthetases and tRNA modification	-0.766
A1618	AA transport and metabolism	Hypothetical	NA	-0.761
A1703	AA transport and metabolism	Energy Metabolism	Amino acids and amines	-0.759
A2018	General	Hypothetical	NA	-0.758
A1427	Coenzyme transport and metabolism	Biosynthesis of cofactors, prosthetic groups, and carriers	Pantothenate	-0.757
A0483	General	Hypothetical	NA	-0.751

Table S9: **Top ten Pearson correlation coefficients between gene transcripts and growth rates for 11 conditions, Related to Figures 5 and S1.** The highest correlation coefficients are given by genes relating to photosynthesis and energy metabolism or amino acid biosynthesis and protein assembly.

**Biomass - ATP maintenance**			
Reaction	Description	Subsystem	PCC
SUCD1Itlm	succinate dehydrogenase	Carbohydrate Metabolism	0.683
SUCD1Icpm	succinate dehydrogenase	Carbohydrate Metabolism	0.683
ME2	malic enzyme (NADP)	Carbohydrate Metabolism and Energy Metabolism	0.683
CA2T3	Ca2 transport via ion channels	Transport	0.605
GLNS	glutamine synthetase	AA Carbohydrate and Energy Metabolism	0.605
CA2T2	calcium transport out via proton antiport	Transport	0.605
EX_NH4_E	ammonia exchange	Exchange Reaction	0.536
NH4T	ammonium transport via diffusion	Transport	0.536
FUMH	fumarase	AA Metabolism	0.527
GLUSYM	L-Glutamate transport in via sodium symport	Transport	0.519
EX_GLU_E	glutamate exchange	Exchange Reaction	0.519
EX_H2O_E	water exchange	Exchange Reaction	-0.524
H2OT5	H2O transport via diffusion	Transport	-0.524
NADPHPQ9cpm	NADPH dehydrogenase (plastoquinone-9 and 4 protons)	Energy Metabolism	-0.391
ALAD_LR	L-alanine dehydrogenase (reversible)	AA Metabolism	-0.388
LDH_D	D-lactate dehydrogenase	Carbohydrate Metabolism	-0.381
lac_d2	D-lactate transport	Transport	-0.381
EX_lac_d_E	D-lactate exchange	Exchange Reaction	-0.381
NADPHPQ9tlm	NADPH dehydrogenase (plastoquinone-9 and 4 protons)	Energy Metabolism	-0.376
THD2	NAD(P) transhydrogenase	Metabolism of cofactors and vitamins	-0.353
ASPT	L-aspartase	AA Metabolism	-0.324

Table S10: **Top ten Pearson correlation coefficients between Biomass - ATP maintenance fluxes (x) and growth rates (y), Related to Figures 5 and S2.** The flux rates used to calculate PCC were absolute values of the fluxes calculated during bi-level regularized FBA. Reactions that yield the highest positive correlation coefficients belong to pathways relating to amino acid, carbohydrate, exchange and transport metabolism whereas reactions involved in nucleotide metabolism are negatively correlated with the growth rate.

Biomass - Photosystem I			
Reaction	Description	Subsystem	PCC
SUCCt2b	Succinate efflux via proton symport	Transport	0.378
EX_succ_E	Succinate exchange	Exchange	0.378
ASPT	L-aspartase	AA Metabolism	0.364
HDH2	Bidirectional Hydrogenase (NADP/NADPH)	Hydrogen Metabolism	0.355
NADPHPQ9tlm	NADPH dehydrogenase (plastoquinone-9 and 4 protons)	Energy Metabolism	0.349
NAT3	sodium transport out via proton antiport	Transport	0.343
GLUSYM	L-Glutamate transport in via sodium symport	Transport	0.343
EX_GLU_E	glutamate exchange	Exchange	0.343
SSALY	succinate-semialdehyde dehydrogenase (NADP)	AA Metabolism and Carbohydrate Metabolism	0.343
2OGDC	2-oxoglutarate decarboxylase	Exchange	0.343
FDNOR1	ferredoxin-NADP reductase	Energy Metabolism	-0.427
ME2	malic enzyme (NADP)	Carbohydrate Metabolism and Energy Metabolism	-0.259
GAPD_NADP	glyceraldehyde-3-phosphate dehydrogenase (NADP) (phosphorylating)	Energy Metabolism	-0.252
ACONT	aconitase	Carbohydrate Metabolism	-0.246
CS	citrate synthase	Carbohydrate Metabolism	-0.246
ICDHY	isocitrate dehydrogenase (NADP)	Metabolism of other amino acids and Energy Metabolism	-0.246
P5CD	1-pyrroline-5-carboxylate dehydrogenase	AA Metabolism	-0.242
FDPQ	Cyclic reaction (ferredoxin:plastoquinol)	Energy Metabolism	-0.240
CA2ABC1	calcium efflux via ABC system	Transport	-0.239
COABC	Cobalt transport via ABC system	Transport	-0.239
DADPEP	D-ala-D-ala dipeptidase	AA Metabolism	-0.239
DAGK_SYN	diacylglycerol kinase (Synechococcus)	Lipid metabolism	-0.239
EX_PI_E	phosphate exchange	Exchange Reaction	-0.239
FTHFCL	5-formyltetrahydrofolate cyclo-ligase	Metabolism of cofactors and vitamins	-0.239
NTD7	5”-nucleotidase (AMP)	Nucleotide Metabolism	-0.239
PIABC	phosphate transport via ABC system	Transport	-0.239
THFAT	tetrahydrofolate aminomethyltransferase	Metabolism of cofactors and vitamins	-0.239
DAGPYP_SYN	diacylglycerol pyrophosphate phosphatase (Synechococcus)	Fatty Acid Synthesis	-0.239
ILEDIFF	L-isoleucine transport out via diffusion	Transport	-0.239
LEUDIFF	L-leucine transport out via diffusion	Transport	-0.239
VALDIFF	L-valine transport out via diffusion	Transport	-0.239

Table S11: **Top ten Pearson correlation coefficients between Biomass- Photosystem I fluxes (x) and growth rates (y), Related to Figures 5 and S3.**

Biomass - Photosystem II			
Reaction	Description	Subsystem	PCC
SUCCt2b	Succinate efflux via proton symport	Transport	0.373
EX_succ_E	Succinate exchange	Exchange	0.373
ASPT	L-aspartase	AA Metabolism	0.364
NADPHPQ9tlm	NADPH dehydrogenase (plastoquinone-9 and 4 protons)	Energy Metabolism	0.354
HDH2	Bidirectional Hydrogenase (NADP/NADPH)	Hydrogen Metabolism	0.354
NAT3	sodium transport out via proton antiport	Transport	0.344
GLUSYM	L-Glutamate transport in via sodium symport	Transport	0.343
EX_GLU_E	glutamate exchange	Exchange	0.343
SSALY	succinate-semialdehyde dehydrogenase (NADP)	AA Metabolism and Carbohydrate Metabolism	0.342
2OGDC	2-oxoglutarate decarboxylase	Exchange	0.342
PSIIR	photosystem II reaction	Energy Metabolism	-0.319
O2EXtll	oxygen exchange between thylakoid lumen and cytosol	None	-0.319
H2OEXtll	water exchange between thylakoid lumen and cytosol	None	-0.319
PFK	phosphofructokinase	Carbohydrate Metabolism	-0.239
FRUK	fructokinase	Carbohydrate Metabolism	-0.239
SPS	sucrose-phosphate synthase	Carbohydrate Metabolism	-0.239
SUCPPT	sucrose-phosphate phosphatase	Carbohydrate Metabolism	-0.239
SUCPR	sucrose phosphorylase	Carbohydrate Metabolism	-0.239
GLCP	glycogen phosphorylase	Carbohydrate Metabolism	-0.239
CTPD	CTP deaminase	Nucleotide Metabolism	-0.239

Table S12: **Top ten Pearson correlation coefficients between fluxes for Biomass - Photosystem II (x) and growth rates (y), Related to Figures 5 and S4.**

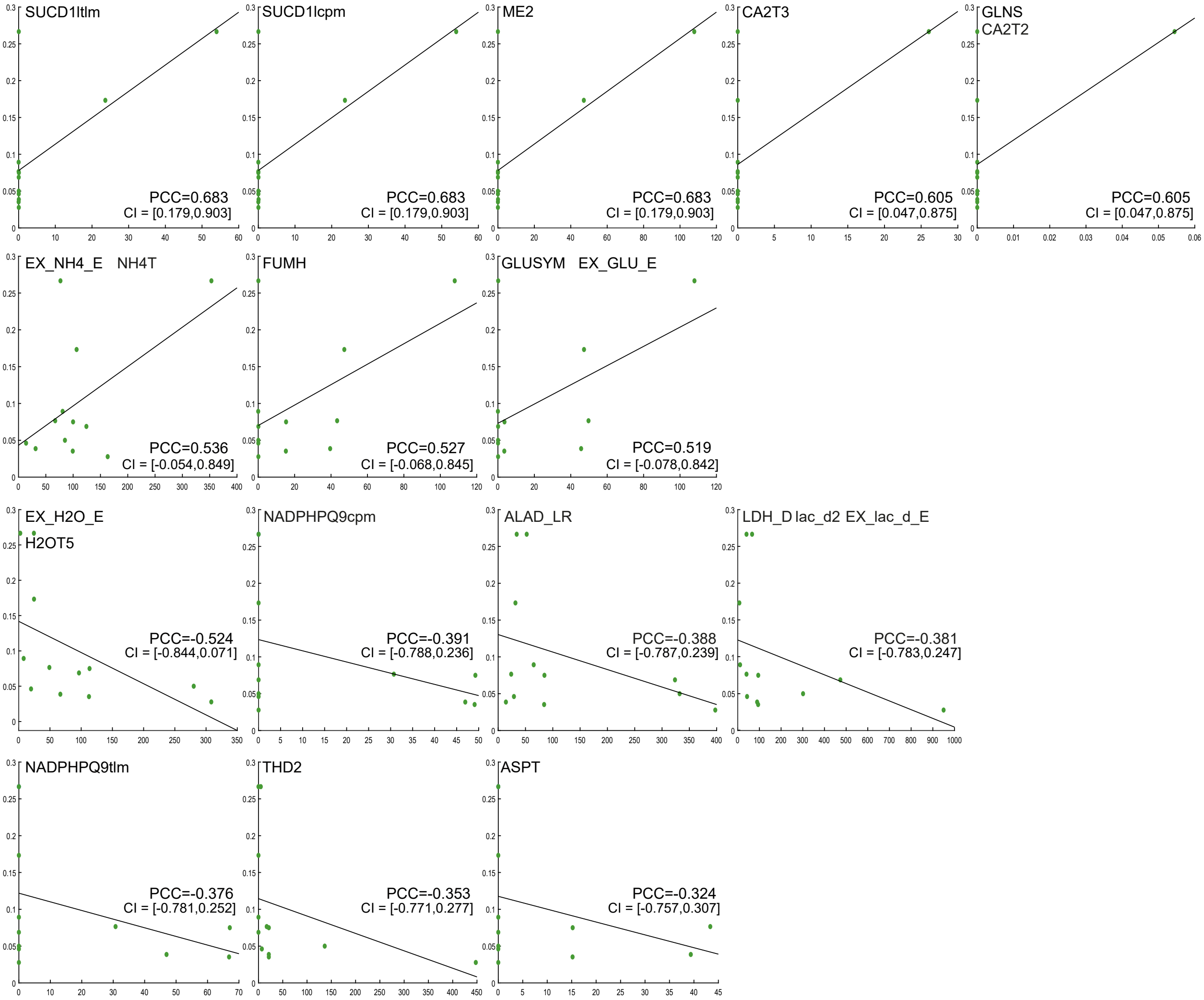

Figure S2: **Top ten Pearson correlation coefficients (PCC) with their respective 95% confidence intervals (CI) between fluxes obtained using Biomass - ATP maintenance as objective functions (x) and growth rates (y), Related to Figure 5.** A list of each reaction and its respective PCC is provided in Table S10.

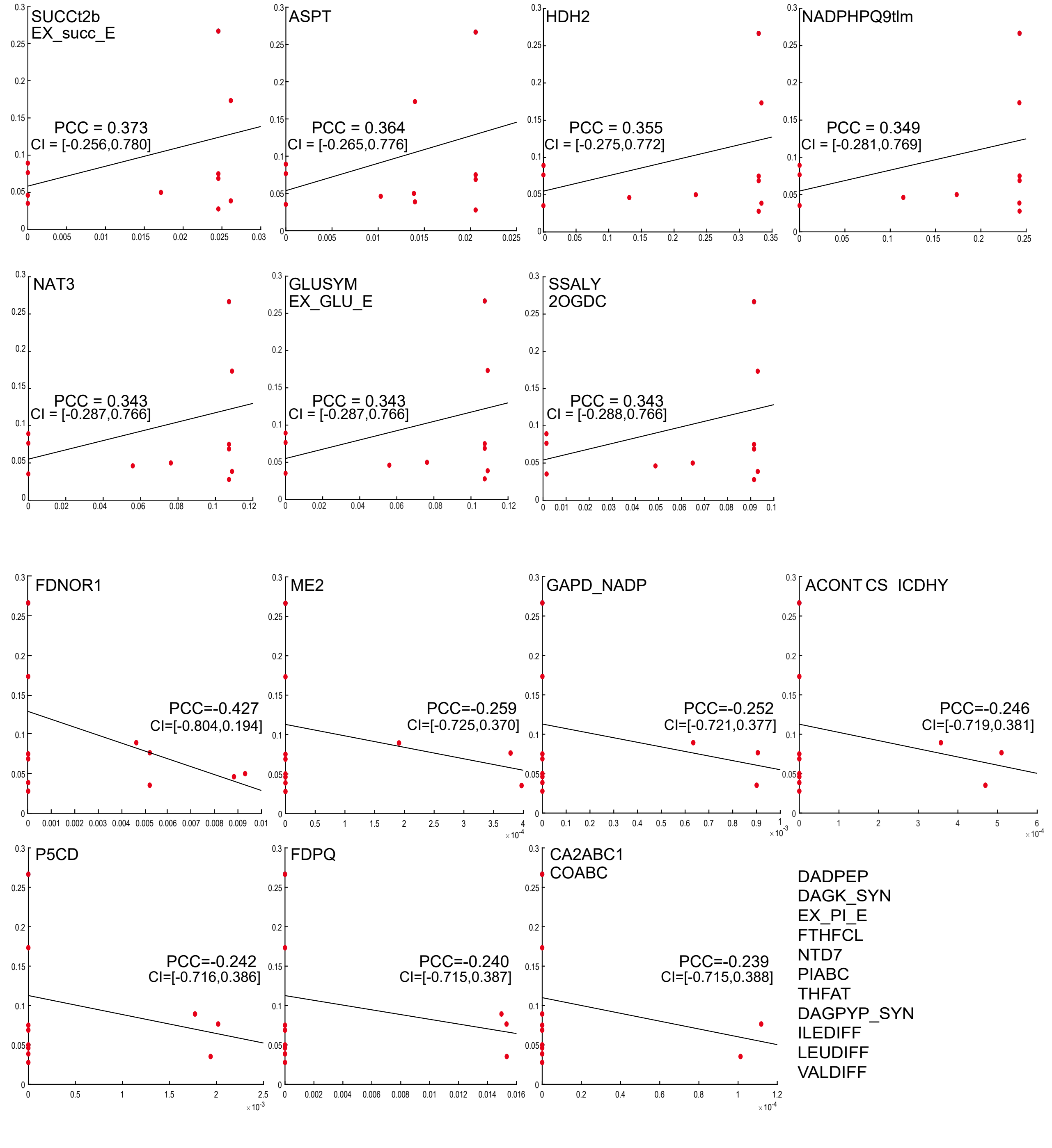

Figure S3: **Top ten Pearson correlation coefficients (PCC) with their respective 95% confidence intervals (CI) between fluxes obtained using Biomass - Photosystem I as objective functions (x) and growth rates (y), Related to Figure 5.** A list of each reaction and its respective PCC is provided in Table S11.

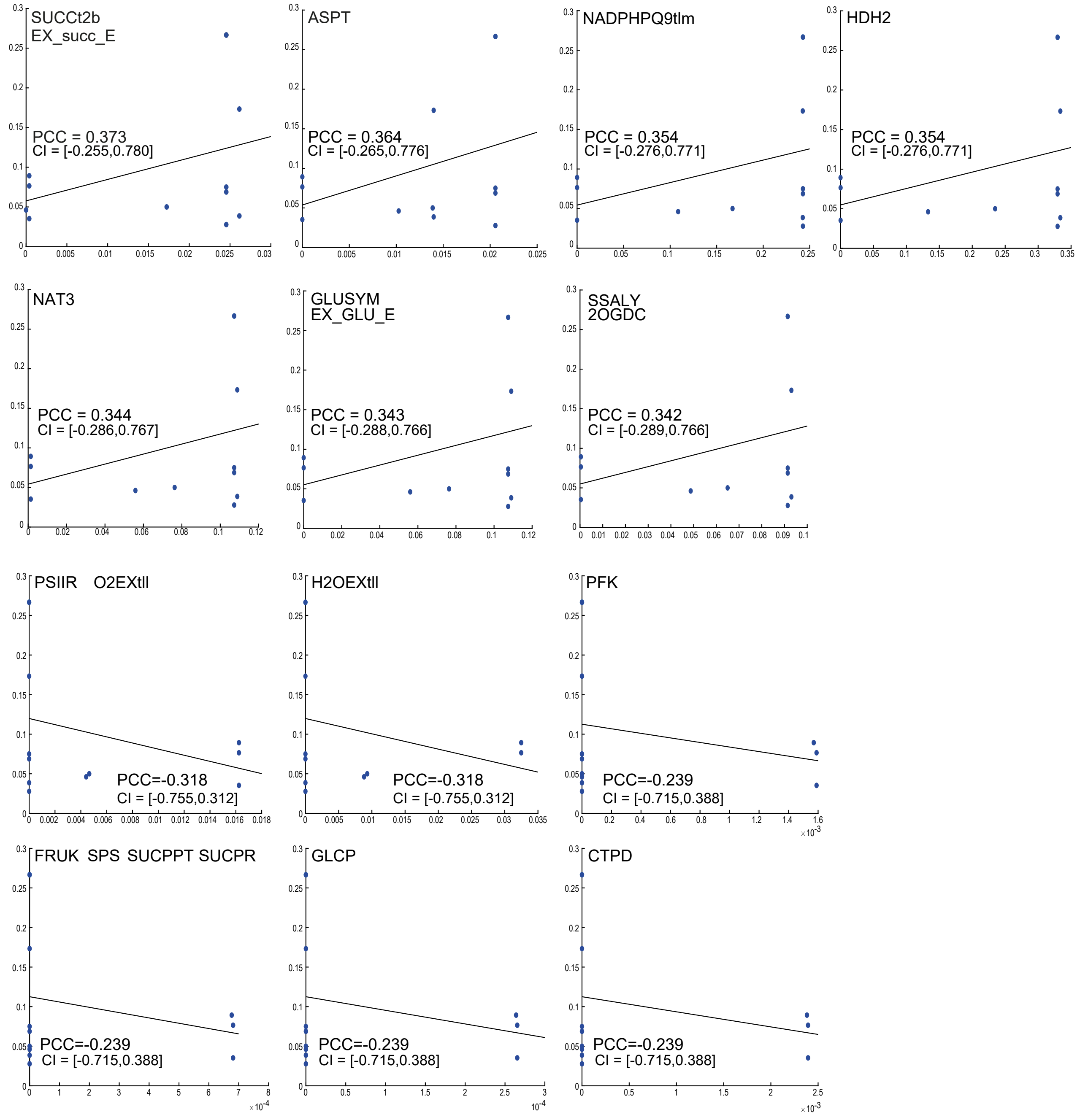

Figure S4: **Top ten Pearson correlation coefficients (PCC) with their respective 95% confidence intervals (CI) between fluxes obtained using Biomass - Photosystem II as objective functions (x) and growth rates (y), Related to Figure 5.** A list of each reaction and its respective PCC is provided in Table S12.

Biomass - ATP maintenance

Gene	COG category	CY category	CY subcategory	PCC
A0639	None	Photosynthesis and respiration	Phycobilisome	0.874
A2457	Post-translational modification, protein turnover, chaperones	Cellular processes	Chaperones	0.828
A1008	None	Photosynthesis and respiration	Photosystem I	0.821
E0022	None	Unknown	NA	0.811
A0561	None	Hypothetical	NA	0.770
A0575	None	Hypothetical	NA	0.767
A2725	None	NA	NA	0.759
A1802	Energy production and conversion/Secondary metabolites biosynthesis, transport and metabolism	Photosynthesis and respiration	CO_2 fixation	0.742
A0881	AA transport and metabolism	AA biosynthesis	Aspartate family	0.734
A0472	None	Hypothetical	NA	0.727
G0060	Inorganic ion transport and metabolism	Transport and binding proteins	NA	-0.835
A2485	General	Hypothetical	NA	-0.831
A0356	General	Translation	Degradation of proteins peptides and glycopeptides	-0.787
A1839	None	Other	Other	-0.781
A1133	Replication, recombination, and repair	Translation	Aminoacyl tRNA synthetases and tRNA modification	-0.766
A1618	AA transport and metabolism	Hypothetical	NA	-0.761
A1703	AA transport and metabolism	Energy Metabolism	Amino acids and amines	-0.759
A2018	General	Hypothetical	NA	-0.758
A1427	Coenzyme transport and metabolism	Biosynthesis of cofactors, prosthetic groups, and carriers	Pantothenate	-0.757
A0483	General	Hypothetical	NA	-0.751

Biomass - Photosystem I

Gene	COG category	CY category	CY subcategory	PCC
A0639	None	Photosynthesis and respiration	Phycobilisome	0.874
A2457	Post-translational modification, protein turnover, chaperones	Cellular processes	Chaperones	0.828
A1008	None	Photosynthesis and respiration	Photosystem I	0.821
E0022	None	Unknown	NA	0.811
A0561	None	Hypothetical	NA	0.770
A0575	None	Hypothetical	NA	0.767
A2725	None	NA	NA	0.759
A1802	Energy production and conversion/Secondary metabolites biosynthesis, transport and metabolism	Photosynthesis and respiration	CO_2 fixation	0.742
A0881	AA transport and metabolism	AA biosynthesis	Aspartate family	0.734
A0472	None	Hypothetical	NA	0.727
G0060	Inorganic ion transport and metabolism	Transport and binding proteins	NA	-0.835
A2485	General	Hypothetical	NA	-0.831
A0356	General	Translation	Degradation of proteins peptides and glycopeptides	-0.787
A1839	None	Other	Other	-0.781
A1133	Replication, recombination, and repair	Translation	Aminoacyl tRNA synthetases and tRNA modification	-0.766
A1618	AA transport and metabolism	Hypothetical	NA	-0.761
A1703	AA transport and metabolism	Energy Metabolism	Amino acids and amines	-0.759
A2018	General	Hypothetical	NA	-0.758
A1427	Coenzyme transport and metabolism	Biosynthesis of cofactors, prosthetic groups, and carriers	Pantothenate	-0.757
A0483	General	Hypothetical	NA	-0.751

Biomass - Photosystem II

Gene	COG category	CY category	CY subcategory	PCC
A0639	None	Photosynthesis and respiration	Phycobilisome	0.874
A2457	Post-translational modification, protein turnover, chaperones	Cellular processes	Chaperones	0.828
A1008	None	Photosynthesis and respiration	Photosystem I	0.821
E0022	None	Unknown	NA	0.811
A0561	None	Hypothetical	NA	0.770
A0575	None	Hypothetical	NA	0.767
A2725	None	NA	NA	0.759
A1802	Energy production and conversion/Secondary metabolites biosynthesis, transport and metabolism	Photosynthesis and respiration	CO_2 fixation	0.742
A0881	AA transport and metabolism	AA biosynthesis	Aspartate family	0.734
A0472	None	Hypothetical	NA	0.727
G0060	Inorganic ion transport and metabolism	Transport and binding proteins	NA	-0.835
A2485	General	Hypothetical	NA	-0.831
A0356	General	Translation	Degradation of proteins peptides and glycopeptides	-0.787
A1839	None	Other	Other	-0.781
A1133	Replication, recombination, and repair	Translation	Aminoacyl tRNA synthetases and tRNA modification	-0.766
A1618	AA transport and metabolism	Hypothetical	NA	-0.761
A1703	AA transport and metabolism	Energy Metabolism	Amino acids and amines	-0.759
A2018	General	Hypothetical	NA	-0.758
A1427	Coenzyme transport and metabolism	Biosynthesis of cofactors, prosthetic groups, and carriers	Pantothenate	-0.757
A0483	General	Hypothetical	NA	-0.751

Table S13: **Top ten Pearson correlation coefficients between transcript-and-flux data (x) and growth rates (y), Related to Figures 5 and S1.** The same genes are already highlighted in Table S9.

References

Aikawa, S., Nishida, A., Ho, S.H., Chang, J.S., Hasunuma, T., Kondo, A., 2014. Glycogen production for biofuels by the euryhaline cyanobacteria *Synechococcus* sp. strain pcc 7002 from an oceanic environment. Biotechnology for biofuels 7, 88.

Angione, C., Conway, M., Lió, P., 2016. Multiplex methods provide effective integration of multi-omic data in genome-scale models. BMC bioinformatics 17, 257.

Angione, C., Lió, P., 2015. Predictive analytics of environmental adaptability in multi-omic network models. Scientific reports 5, 15147.

Bartsevich, V.V., Pakrasi, H., 1995. Molecular identification of an abc transporter complex for manganese: analysis of a cyanobacterial mutant strain impaired in the photosynthetic oxygen evolution process. The EMBO journal 14, 1845–1853.

Boulay, C., Abasova, L., Six, C., Vass, I., Kirilovsky, D., 2008. Occurrence and function of the orange carotenoid protein in photoprotective mechanisms in various cyanobacteria. Biochimica et Biophysica Acta (BBA)-Bioenergetics 1777, 1344–1354.

Checchetto, V., Segalla, A., Allorent, G., La Rocca, N., Leanza, L., Giacometti, G.M., Uozumi, N., Finazzi, G., Bergantino, E., Szabò, I., 2012. Thylakoid potassium channel is required for efficient photosynthesis in cyanobacteria. Proceedings of the National Academy of Sciences 109, 11043–11048.

Clark, R.L., McGinley, L.L., Purdy, H.M., Korosh, T.C., Reed, J.L., Root, T.W., Pfleger, B.F., 2018. Light-optimized growth of cyanobacterial cultures: Growth phases and productivity of biomass and secreted molecules in light-limited batch growth. Metabolic engineering 47, 230–242.

Culley, C., Vijayakumar, S., Zampieri, G., Angione, C., 2020. A mechanism-aware and multiomic machine-learning pipeline characterizes yeast cell growth. Proceedings of the National Academy of Sciences 117, 18869–18879.

Demtröder, L., Narberhaus, F., Masepohl, B., 2019. Coordinated regulation of nitrogen fixation and molybdate transport by molybdenum. Molecular microbiology 111, 17–30.

Ebrahim, A., Brunk, E., Tan, J., O'brien, E.J., Kim, D., Szubin, R., Lerman, J.A., Lechner, A., Sastry, A., Bordbar, A., et al., 2016. Multi-omic data integration enables discovery of hidden biological regularities. Nature communications 7, 1–9.

Flores, E., Frías, J.E., Rubio, L.M., Herrero, A., 2005. Photosynthetic nitrate assimilation in cyanobacteria. Photosynthesis Research 83, 117–133.

Fujisawa, T., Narikawa, R., Maeda, S.i., Watanabe, S., Kanesaki, Y., Kobayashi, K., Nomata, J., Hanaoka, M., Watanabe, M., Ehira, S., et al., 2016. Cyanobase: a large-scale update on its 20th anniversary. Nucleic acids research 45, D551–D554.

Heikal, A., Nakatani, Y., Dunn, E., Weimar, M.R., Day, C.L., Baker, E.N., Lott, J.S., Sazanov, L.A., Cook, G.M., 2014. Structure of the bacterial type ii nadh dehydrogenase: a monotopic membrane protein with an essential role in energy generation. Molecular microbiology 91, 950–964.

Heirendt, L., Arreckx, S., Pfau, T., Mendoza, S.N., Richelle, A., Heinken, A., Haraldsdóttir, H.S., Wachowiak, J., Keating, S.M., Vlasov, V., et al., 2019. Creation and analysis of biochemical constraint-based models using the cobra toolbox v. 3.0. Nature protocols 14, 639–702.

Hendry, J.I., Prasannan, C.B., Joshi, A., Dasgupta, S., Wangikar, P.P., 2016. Metabolic model of *Synechococcus* sp. pcc 7002: Prediction of flux distribution and network modification for enhanced biofuel production. Bioresource Technology 213, 190–197.

Huertas, M.J., López-Maury, L., Giner-Lamia, J., Sánchez-Riego, A.M., Florencio, F.J., 2014. Metals in cyanobacteria: analysis of the copper, nickel, cobalt and arsenic homeostasis mechanisms. Life 4, 865–886.

Jackson, S.A., Eaton-Rye, J.J., Bryant, D.A., Posewitz, M.C., Davies, F.K., 2015. Dynamics of photosynthesis in a glycogen-deficient glgc mutant of *Synechococcus* sp. strain pcc 7002. Appl. Environ. Microbiol. 81, 6210–6222.

Kato, A., Takatani, N., Ikeda, K., Maeda, S.i., Omata, T., 2017. Removal of the product from the culture medium strongly enhances free fatty acid production by genetically engineered *Synechococcus elongatus*. Biotechnology for biofuels 10, 141.

Kim, J., Fabris, M., Baart, G., Kim, M.K., Goossens, A., Vyverman, W., Falkowski, P.G., Lun, D.S., 2016. Flux balance analysis of primary metabolism in the diatom phaeodactylum tricornutum. The Plant Journal 85, 161–176.

Lê, S., Josse, J., Husson, F., et al., 2008. Factominer: an r package for multivariate analysis. Journal of statistical software 25, 1–18.

Lea-Smith, D.J., Bombelli, P., Vasudevan, R., Howe, C.J., 2016. Photosynthetic, respiratory and extracellular electron transport pathways in cyanobacteria. Biochimica et Biophysica Acta (BBA)-Bioenergetics 1857, 247–255.

Liu, X., Yang, M., Wang, Y., Chen, Z., Zhang, J., Lin, X., Ge, F., Zhao, J., 2018. Effects of psii manganese-stabilizing protein succinylation on photosynthesis in the model cyanobacterium *Synechococcus* sp. pcc 7002. Plant and Cell Physiology 59, 1466–1482.

Ludwig, M., Bryant, D.A., 2011. Transcription profiling of the model cyanobacterium *Synechococcus* sp. strain pcc 7002 by next-gen (solid™) sequencing of cdna. Frontiers in microbiology 2, 41.

Ludwig, M., Bryant, D.A., 2012a. Acclimation of the global transcriptome of the cyanobacterium *Synechococcus* sp. strain pcc 7002 to nutrient limitations and different nitrogen sources. Frontiers in microbiology 3, 145.

Ludwig, M., Bryant, D.A., 2012b. *Synechococcus* sp. strain pcc 7002 transcriptome: acclimation to temperature, salinity, oxidative stress, and mixotrophic growth conditions. Frontiers in microbiology 3, 354.

McConnell, M.D., Cowgill, J.B., Baker, P.L., Rappaport, F., Redding, K.E., 2011. Double reduction of plastoquinone to plastoquinol in photosystem 1. Biochemistry 50, 11034–11046.

McNeely, K., Xu, Y., Bennette, N., Bryant, D.A., Dismukes, G.C., 2010. Redirecting reductant flux into hydrogen production via metabolic engineering of fermentative carbon metabolism in a cyanobacterium. Applied and environmental microbiology 76, 5032–5038.

Myers, J.A., Curtis, B.S., Curtis, W.R., 2013. Improving accuracy of cell and chromophore concentration measurements using optical density. BMC biophysics 6, 4.

Noreña-Caro, D., Benton, M.G., 2018. Cyanobacteria as photoautotrophic biofactories of high-value chemicals. Journal of CO2 Utilization 28, 335–366.

Palenik, B., Brahamsha, B., Larimer, F., Land, M., Hauser, L., Chain, P., Lamerdin, J., Regala, W., Allen, E., McCarren, J., et al., 2003. The genome of a motile marine *Synechococcus*. Nature 424, 1037–1042.

Peltier, G., Aro, E.M., Shikanai, T., 2016. Ndh-1 and ndh-2 plastoquinone reductases in oxygenic photosynthesis. Annual review of plant biology 67, 55–80.

Qian, X., Kumaraswamy, G.K., Zhang, S., Gates, C., Ananyev, G.M., Bryant, D.A., Dismukes, G.C., 2016. Inactivation of nitrate reductase alters metabolic branching of carbohydrate fermentation in the cyanobacterium *Synechococcus* sp. strain pcc 7002. Biotechnology and bioengineering 113, 979–988.

Qiao, C., Duan, Y., Zhang, M., Hagemann, M., Luo, Q., Lu, X., 2018. Effects of reduced and enhanced glycogen pools on salt-induced sucrose production in a sucrose-secreting strain of *Synechococcus elongatus* pcc 7942. Appl. Environ. Microbiol. 84, e02023–17.

Saha, R., Liu, D., Hoynes-O'Connor, A., Liberton, M., Yu, J., Bhattacharyya-Pakrasi, M., Balassy, A., Zhang, F., Moon, T.S., Maranas, C.D., et al., 2016. Diurnal regulation of cellu-

lar processes in the cyanobacterium *Synechocystis* sp. strain pcc 6803: Insights from transcriptomic, fluxomic, and physiological analyses. MBio 7, e00464–16.

Sarkar, D., Mueller, T.J., Liu, D., Pakrasi, H.B., Maranas, C.D., 2019. A diurnal flux balance model of *Synechocystis* sp. pcc 6803 metabolism. PLOS Computational Biology 15, e1006692.

Shcolnick, S., Keren, N., 2006. Metal homeostasis in cyanobacteria and chloroplasts. balancing benefits and risks to the photosynthetic apparatus. Plant physiology 141, 805–810.

Shvarev, D., Maldener, I., 2018. Atp-binding cassette transporters of the multicellular cyanobacterium anabaena sp. pcc 7120: A wide variety for a complex lifestyle. FEMS microbiology letters 365, fny012.

Ueda, K., Nakajima, T., Yoshikawa, K., Toya, Y., Matsuda, F., Shimizu, H., 2018. Metabolic flux of the oxidative pentose phosphate pathway under low light conditions in *Synechocystis* sp. pcc 6803. Journal of bioscience and bioengineering 126, 38–43.

Vu, T.T., Stolyar, S.M., Pinchuk, G.E., Hill, E.A., Kucek, L.A., Brown, R.N., Lipton, M.S., Osterman, A., Fredrickson, J.K., Konopka, A.E., et al., 2012. Genome-scale modeling of light-driven reductant partitioning and carbon fluxes in diazotrophic unicellular cyanobacterium *Cyanothece* sp. atcc 51142. PLoS computational biology 8, e1002460.

Watanabe, M., Semchonok, D.A., Webber-Birungi, M.T., Ehira, S., Kondo, K., Narikawa, R., Ohmori, M., Boekema, E.J., Ikeuchi, M., 2014. Attachment of phycobilisomes in an antenna–photosystem i supercomplex of cyanobacteria. Proceedings of the National Academy of Sciences 111, 2512–2517.

Woo, J.E., Jang, Y.S., 2019. Metabolic engineering of microorganisms for the production of ethanol and butanol from oxides of carbon. Applied Microbiology and Biotechnology 103, 8283–8292.

Yang, J.H., Wright, S.N., Hamblin, M., McCloskey, D., Alcantar, M.A., Schrübbers, L., Lopatkin, A.J., Satish, S., Nili, A., Palsson, B.O., et al., 2019. A white-box machine learning approach for revealing antibiotic mechanisms of action. Cell 177, 1649–1661.

Yang, Y., Feng, J., Li, T., Ge, F., Zhao, J., 2015. Cyanomics: an integrated database of omics for the model cyanobacterium *Synechococcus* sp. pcc 7002. Database 2015, bau127.

Zampieri, G., Vijayakumar, S., Yaneske, E., Angione, C., 2019. Machine and deep learning meet genome-scale metabolic modeling. PLoS computational biology 15.

Zhang, J., Petersen, S.D., Radivojevic, T., Ramirez, A., Pérez-Manríquez, A., Abeliuk, E., Sánchez, B.J., Costello, Z., Chen, Y., Fero, M.J., et al., 2020. Combining mechanistic and machine learning models for predictive engineering and optimization of tryptophan metabolism. Nature Communications 11, 1–13.
